# The Impact of COVID-19 on RNA Therapeutics: A Surge in Lipid Nanoparticles and Alternative Delivery Systems

**DOI:** 10.3390/pharmaceutics16111366

**Published:** 2024-10-25

**Authors:** Nargish Parvin, Tapas K. Mandal, Sang-Woo Joo

**Affiliations:** School of Mechanical Engineering, School of Basic Science, Yeungnam University, Gyeongsan 38541, Republic of Korea; nargish.parvin@gmail.com

**Keywords:** RNA therapeutics, lipid nanoparticles, COVID-19 vaccines, drug delivery systems, mRNA technology

## Abstract

The COVID-19 pandemic has significantly accelerated progress in RNA-based therapeutics, particularly through the successful development and global rollout of mRNA vaccines. This review delves into the transformative impact of the pandemic on RNA therapeutics, with a strong focus on lipid nanoparticles (LNPs) as a pivotal delivery platform. LNPs have proven to be critical in enhancing the stability, bioavailability, and targeted delivery of mRNA, facilitating the unprecedented success of vaccines like those developed by Pfizer-BioNTech and Moderna. Beyond vaccines, LNP technology is being explored for broader therapeutic applications, including treatments for cancer, rare genetic disorders, and infectious diseases. This review also discusses emerging RNA delivery systems, such as polymeric nanoparticles and viral vectors, which offer alternative strategies to overcome existing challenges related to stability, immune responses, and tissue-specific targeting. Additionally, we examine the pandemic’s influence on regulatory processes, including the fast-tracked approvals for RNA therapies, and the surge in research funding that has spurred further innovation in the field. Public acceptance of RNA-based treatments has also grown, laying the groundwork for future developments in personalized medicine. By providing an in-depth analysis of these advancements, this review highlights the long-term impact of COVID-19 on the evolution of RNA therapeutics and the future of precision drug delivery technologies.

## 1. Introduction

### 1.1. Overview of RNA Therapeutics

**Figure 1:**
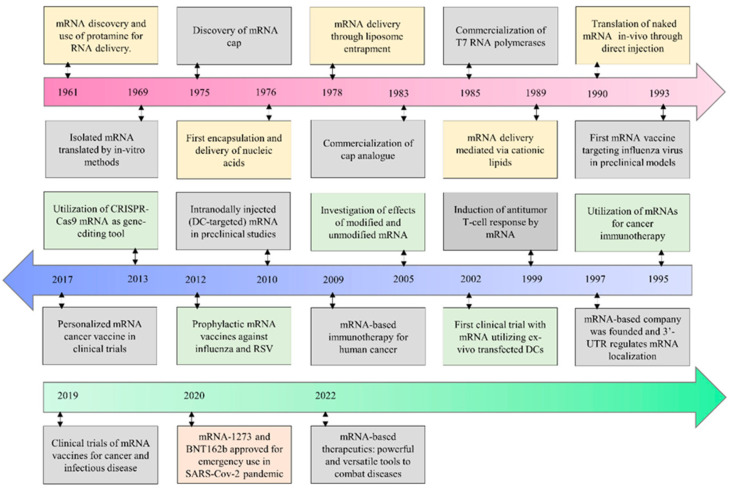
Key historical milestones in the development of mRNA-based therapeutics. Reprinted with permission from Ref. [6] under the Creative Commons Attribution 4.0 International License.

RNA therapeutics has emerged as a groundbreaking class of treatments designed to manipulate gene expression for therapeutic benefit. Unlike traditional small molecules that target proteins, RNA-based therapies intervene directly at the level of gene expression. This includes messenger RNA (mRNA) therapies, which can replace or supplement proteins by instructing cells to synthesize them, as well as small interfering RNA (siRNA) and antisense oligonucleotides (ASOs), which are designed to silence genes by degrading mRNA or blocking its translation. The potential of RNA therapeutics spans a range of applications, including genetic disorders, cancer, infectious diseases, and metabolic conditions. The primary advantage of RNA-based therapeutics lies in their ability to modulate gene expression with a high degree of specificity. By targeting the genetic root of diseases rather than just the resulting protein products, RNA therapies offer a powerful means of treating conditions that are otherwise considered untreatable or incurable [1,2]. Despite their promise, RNA therapies have historically faced significant challenges, particularly concerning their delivery and stability. RNA molecules are inherently unstable due to their susceptibility to enzymatic degradation, and delivering them into cells without triggering immune responses has posed major hurdles. However, breakthroughs in delivery technologies—most notably lipid nanoparticles (LNPs)—have significantly advanced the field. LNPs encapsulate RNA molecules, protecting them from degradation and facilitating their uptake into target cells [3]. One of the first successful RNA-based therapies, patisiran (an siRNA drug), received FDA approval in 2018 for the treatment of hereditary transthyretin-mediated amyloidosis. This marked a major milestone in the field, demonstrating the viability of RNA as a therapeutic modality. Other RNA therapies, such as ASOs for spinal muscular atrophy (nusinersen), have followed, further solidifying the clinical relevance of RNA-based treatments [4,5]. The rapid advancement of RNA therapeutics has been further accelerated by global events, particularly the COVID-19 pandemic. Figure 1 highlights the key milestones in the evolution of mRNA-based therapeutics, tracing its development from early conceptual studies to its modern applications [6]. The timeline demonstrates the initial breakthroughs in understanding mRNA synthesis, modifications to enhance stability, and the eventual success of lipid nanoparticle (LNP) technology for efficient delivery. Significant advances, such as the first FDA-approved mRNA vaccine, underscore the progress made in utilizing mRNA for both vaccines and therapeutic applications. These milestones collectively illustrate the rapid development and growing impact of mRNA technology in medicine.

### 1.2. The Role of COVID-19 in Advancing RNA Technologies

The COVID-19 pandemic was a pivotal moment for RNA therapeutics, as it catapulted mRNA vaccines into the global spotlight. Prior to the pandemic, RNA vaccines had been under investigation for several years, primarily in the context of cancer immunotherapy and vaccines against infectious diseases. However, their widespread adoption has been limited due to concerns over stability, immune activation, and efficient delivery. The emergence of SARS-CoV-2 in 2019 prompted a rapid acceleration of RNA vaccine research, culminating in the approval of two mRNA-based COVID-19 vaccines—produced by Pfizer-BioNTech (BNT162b2) and Moderna (mRNA-1273)—in record time [7]. The success of mRNA vaccines during the COVID-19 pandemic can largely be attributed to the use of lipid nanoparticles as a delivery system. LNPs, composed of ionizable lipids, cholesterol, and other components, enable the safe and efficient delivery of mRNA into cells. They protect the mRNA from degradation by nucleases and facilitate its entry into cells, where they instruct the production of viral spike proteins, eliciting an immune response [8]. The widespread use of LNPs in these vaccines highlighted their potential as a delivery system for RNA therapeutics beyond infectious diseases, opening doors to a range of applications in areas such as oncology, rare diseases, and regenerative medicine. The pandemic also underscored the scalability and rapid adaptability of RNA platforms. Traditional vaccine development typically takes years, but mRNA vaccine candidates for COVID-19 were designed and entered clinical trials within months. This flexibility is a hallmark of RNA platforms, as mRNA sequences can be quickly modified to match emerging viral variants or other disease targets without requiring extensive changes to the underlying delivery technology [9]. The speed at which mRNA vaccines were developed and deployed has set a new precedent for vaccine research and has accelerated the adoption of RNA-based technologies across multiple fields. Beyond mRNA vaccines, the pandemic has spurred interest in alternative RNA delivery systems, including those designed to overcome some of the limitations of LNPs. While LNPs are highly effective, they are not without drawbacks. Some challenges include potential toxicity, immune responses against the delivery vehicle, and difficulty in targeting specific tissues beyond the liver. Researchers are now investigating novel delivery systems, such as polymer-based nanoparticles, exosomes, and peptide-based carriers, which may offer improved targeting capabilities and reduced side effects [10].

### 1.3. Research Questions

The primary aim of this review is to examine the profound impact of the COVID-19 pandemic on the field of RNA therapeutics, with a specific focus on lipid nanoparticles (LNPs) and alternative RNA delivery systems. While LNPs played a crucial role in the success of mRNA vaccines during the pandemic, they are not the only delivery option available. This review will provide a comprehensive overview of how the global urgency to develop RNA-based vaccines and treatments accelerated innovations in RNA delivery technologies. Specifically, we will explore the following Research Questions:

1. How did lipid nanoparticles (LNPs) emerge as the preferred platform for RNA delivery during the COVID-19 pandemic? We will discuss how LNPs enabled the rapid deployment of COVID-19 vaccines and the mechanisms by which they facilitate mRNA delivery. We will also address the advantages and limitations of LNPs and the ongoing research efforts to optimize their use in therapeutic settings [11].
2. What alternative RNA delivery systems have gained attention due to the limitations of LNPs? Although LNPs have proven successful, there is a growing interest in developing alternative delivery platforms that address some of the limitations associated with LNPs, such as tissue specificity, immunogenicity, and scalability. We will review novel approaches, including polymeric nanoparticles, exosomes, and viral vectors, and evaluate their potential to enhance RNA therapeutic efficacy [12].
3. How has the COVID-19 pandemic influenced the long-term development of RNA therapeutics? Finally, this review will consider how the rapid development and widespread success of mRNA vaccines during the COVID-19 pandemic has reshaped the landscape of RNA therapeutics. The pandemic has not only demonstrated the feasibility of RNA-based treatments but also highlighted areas that require further investigation, including delivery challenges and regulatory considerations for next-generation RNA therapies [13]. Therefore, the COVID-19 pandemic has had a transformative impact on the field of RNA therapeutics, driving innovation in delivery systems and setting the stage for the future of gene-based medicine. The lessons learned from the pandemic will continue to influence the development of RNA therapies, making them an increasingly viable option for a wide range of diseases. Through this review, we aim to provide a detailed understanding of the technological advancements in RNA delivery systems and show how they have been shaped by the unprecedented challenges and opportunities presented by the pandemic.

## 2. The Evolution of RNA Therapeutics Pre-COVID-19

### 2.1. Early Developments in RNA-Based Therapies

The development of RNA-based therapies can be traced back to the discovery of RNA interference (RNAi) in the late 1990s. This discovery revolutionized the field of molecular biology by showing that small RNA molecules could selectively inhibit gene expression. The breakthrough led to the concept of using small interfering RNAs (siRNAs) to silence disease-causing genes. The promise of RNAi prompted extensive research and development efforts, and it was soon followed by the exploration of other RNA-based modalities, such as antisense oligonucleotides (ASOs), which could also inhibit gene expression by binding to specific mRNA targets [11].

To date, three small interfering RNA (siRNA) drugs—patisiran, givosiran, and lumasiran—have gained FDA approval, with seven additional siRNA candidates, including inclisiran, vutrisiran, fitusiran, cosdosiran, nedosiran, tivanisiran, and teprasiran, currently in Phase III clinical trials. Patisiran, the first RNA interference (RNAi)-based drug approved by the FDA, is used for the treatment of hereditary transthyretin-mediated (hTTR) amyloidosis with polyneuropathy, marking a significant advancement in the field of RNAi therapeutics [12]. Like inotersen, patisiran (ALN-18328) effectively silences mutated mRNAs by targeting the 3′ untranslated region (UTR) of the TTR gene. To enhance the efficacy of siRNA drugs, Alnylam Pharmaceuticals developed a novel delivery platform using GalNAc (N-acetylgalactosamine) conjugation, which is now employed in roughly one-third of siRNA drugs in clinical trials. Revusiran was the first GalNAc-conjugated siRNA designed to increase hepatic uptake by targeting asialoglycoprotein receptors, but it was discontinued following safety concerns during the Phase III ENDEAVOUR trial due to an observed increase in patient mortality [13,14]. Despite this setback, Alnylam continued to refine its GalNAc-conjugation strategies to improve siRNA stability and minimize nuclease degradation. The approval of givosiran and lumasiran by the FDA further validated the safety and efficacy of GalNAc-siRNA drugs, demonstrating their ability to significantly reduce target mRNA levels with minimal adverse effects. Other pharmaceutical companies have also explored fully chemically modified RNAi therapies with increased metabolic stability and structural modifications, extending RNAi applications beyond hepatic diseases to target other organs and conditions, including kidney injury and ocular diseases. For instance, Quark Pharmaceuticals developed QPI-1002 and QPI-1007 to treat kidney injury and eye conditions, respectively. Recently, there has been growing interest in developing RNAi drugs for cancer therapy, such as SiG12D LODER, which delivers KRASG12D siRNA for pancreatic ductal adenocarcinoma treatment, and TKM-080301 and Atu027, which target hepatocellular carcinoma and solid tumors, respectively [15,16,17]. One of the first major successes in the field was the approval of fomivirsen, an antisense drug developed to treat cytomegalovirus retinitis in patients with AIDS. Approved by the FDA in 1998, fomivirsen marked the beginning of RNA-based therapeutic use in the clinic. However, it was not until 2018 that RNA-based therapies truly entered the mainstream with the approval of patisiran, an siRNA-based drug that targets transthyretin (TTR) amyloidosis, a rare genetic disorder [18]. Despite these early advances, RNA-based therapies were not without their challenges. One of the primary obstacles was the instability of RNA in the body. RNases, enzymes that degrade RNA, are abundant in the bloodstream, making it difficult for RNA molecules to reach their intended targets. Additionally, even if RNA molecules could avoid degradation, they needed to be delivered into the cells where their therapeutic effects could be realized. This required innovative delivery systems, which became a major area of focus in RNA therapeutic research [19].

### 2.2. Initial Challenges in RNA Delivery

The early development of RNA therapies faced significant hurdles related to delivery, which were arguably the most significant barrier to clinical success. Naked RNA molecules, when injected into the body, are rapidly degraded by nucleases, making it nearly impossible to achieve therapeutic levels in target tissues. Additionally, RNA molecules are large and negatively charged, which makes it difficult for them to cross the lipid-rich cell membranes to reach the cytoplasm, where their effects are exerted [20]. To overcome these challenges, researchers began exploring various delivery systems. Early strategies included chemical modifications to the RNA backbone to increase stability and reduce recognition by nucleases. For example, phosphorothioate modifications in antisense oligonucleotides replaced a non-bridging oxygen in the phosphate backbone with sulfur, which increased the stability of the oligonucleotides and enhanced their ability to resist degradation in biological fluids [21]. However, chemical modifications alone were not sufficient. A major breakthrough came with the development of lipid-based delivery systems. Lipid nanoparticles (LNPs), which encapsulate RNA molecules within lipid bilayers, provide a means to protect RNA from degradation while also enhancing its cellular uptake. LNPs facilitate the fusion of the lipid bilayer with cell membranes, allowing the encapsulated RNA to be released into the cytoplasm where it can exert its effects [22]. LNPs quickly became a major focus of RNA delivery research, especially for mRNA and siRNA therapeutics, as they offered a practical solution to the instability and delivery challenges that had plagued early RNA therapies. Despite the promise of LNPs, they too were associated with certain challenges. For example, LNPs tend to accumulate in the liver, making them well-suited for liver-targeted therapies but less effective for delivering RNA to other tissues. Furthermore, the immune system can sometimes recognize and respond to LNPs, leading to inflammatory responses that could limit their therapeutic potential. Therefore, while LNPs were a major advancement, further innovation was required to make RNA therapies more broadly applicable across a range of diseases [23].

### 2.3. Pre-Pandemic Innovations in RNA Technology

In the years leading up to the COVID-19 pandemic, several innovations in RNA technology helped pave the way for the rapid development of RNA-based vaccines and therapies. One major advancement was in the field of mRNA vaccines. Traditional vaccine approaches typically involve using inactivated or live-attenuated viruses to elicit an immune response. In contrast, mRNA vaccines work by introducing a piece of synthetic mRNA that encodes for a viral protein, such as the spike protein of a coronavirus, into the body. The body’s cells then produce this protein, which stimulates the immune system to recognize and fight the virus if it is encountered in the future [24]. Although mRNA vaccines had been under development for several years, their potential was not fully realized until the outbreak of COVID-19. Prior to the pandemic, researchers had developed mRNA vaccine candidates for other diseases, including Zika virus and influenza, but these efforts were largely in preclinical stages or early-phase clinical trials. Key innovations in mRNA vaccine development included the use of modified nucleotides to reduce immunogenicity (the tendency of the immune system to react against the mRNA itself) and to increase stability [25]. These advances ensured that mRNA vaccines would remain stable long enough to generate the desired immune response without triggering an unwanted inflammatory reaction. LNPs also played a crucial role in the advancement of mRNA vaccines. The use of LNPs to deliver mRNA was essential for protecting the fragile mRNA molecules from degradation and facilitating their entry into cells. Additionally, LNPs were shown to enhance the immune response generated by mRNA vaccines, making them more effective [26]. Beyond vaccines, pre-pandemic innovations also focused on expanding the range of diseases that could be targeted by RNA therapeutics. One area of particular interest was oncology, where RNA therapies, including mRNA-based cancer vaccines and RNAi-based treatments, were being explored for their ability to modulate the immune system or silence oncogenes. Early trials of these therapies showed promise, although delivery challenges and immune system activation remained key barriers to success [27]. Another notable pre-pandemic innovation was the development of CRISPR-Cas9-based gene editing technologies, which have the potential to be combined with RNA delivery systems. CRISPR, a powerful tool for editing genes, relies on the delivery of RNA molecules that guide the Cas9 enzyme to specific locations in the genome, where it can make precise cuts to modify the DNA. Although CRISPR is not strictly an RNA therapy, the delivery of guide RNAs is an essential component of the technology, and innovations in RNA delivery have thus directly impacted the progress of gene-editing approaches [28]. In the years leading up to the COVID-19 pandemic, these innovations in RNA technology set the stage for a new era of gene-based medicine. Researchers were already exploring the use of RNA therapeutics for a wide range of applications, from rare genetic diseases to common conditions like cancer and cardiovascular disease. However, it was the urgent need for rapid vaccine development during the COVID-19 pandemic that accelerated the adoption of RNA technologies, particularly mRNA vaccines, and brought LNPs and other delivery systems into the global spotlight.

## 3. Impact of COVID-19 on RNA Therapeutics

### 3.1. The Emergence of mRNA Vaccines

The COVID-19 pandemic marked a transformative period for RNA therapeutics, particularly for mRNA-based vaccines. Although mRNA vaccine technology had been under development for several years, its success had been primarily limited to preclinical studies and early-stage clinical trials before 2020. The unprecedented global health crisis created by SARS-CoV-2 catalyzed the rapid development, testing, and deployment of mRNA vaccines, positioning them at the forefront of the fight against the pandemic. The mRNA vaccines developed by Pfizer-BioNTech (BNT162b2) and Moderna (mRNA-1273) were the first to receive emergency use authorizations (EUAs) from regulatory agencies such as the U.S. Food and Drug Administration (FDA) and the European Medicines Agency (EMA). These vaccines encode a modified form of the SARS-CoV-2 spike protein, enabling the host cells to express the protein and generate an immune response that includes neutralizing antibodies and T-cell activation [29]. The swift development and large-scale production of mRNA vaccines were key to controlling the spread of COVID-19 and mitigating its public health impact. One of the significant advantages of mRNA vaccines is the speed with which they can be designed and produced. Traditional vaccine platforms, such as inactivated or live-attenuated viruses, require months to years to develop due to the need for pathogen cultivation, inactivation, and formulation. In contrast, mRNA vaccines can be rapidly synthesized once the genetic sequence of the virus is available. The flexibility of mRNA platforms was demonstrated when mRNA vaccines for COVID-19 entered clinical trials within months of the initial outbreak, a timeline that would have been unthinkable for conventional vaccine approaches [30]. Additionally, mRNA vaccines do not require viral material for production, reducing the biohazard risk associated with vaccine manufacturing and simplifying the scalability of production. This rapid adaptability and scalability are two of the primary reasons mRNA vaccines became the dominant technology for combating the COVID-19 pandemic. Furthermore, the success of these vaccines has spurred interest in applying mRNA technology to other diseases, such as cancer and other infectious diseases, and advancing mRNA-based therapeutic platforms [31].

### 3.2. Lipid Nanoparticles as the Preferred Delivery Vehicle

The success of mRNA vaccines during the COVID-19 pandemic is largely due to the use of lipid nanoparticles (LNPs) as the primary delivery vehicle. LNPs were first developed for delivering small interfering RNAs (siRNAs) and have since been adapted for mRNA delivery. They are composed of a mixture of lipids that form a protective shell around the fragile RNA, preventing its degradation by nucleases and enabling it to enter target cells. This makes LNPs a critical component of the success of RNA therapeutics, as naked RNA is highly unstable in biological environments [32].

The composition of LNPs typically includes four key components: ionizable lipids, cholesterol, phospholipids, and polyethylene glycol (PEG). Each component plays a specific role in the structure and function of LNPs:

1. Ionizable lipids are the most critical element, as they help the LNPs bind to the negatively charged RNA molecules. These lipids become positively charged in an acidic environment, enabling interaction with RNA and facilitating endosomal escape after cellular uptake.
2. Cholesterol adds stability to the LNPs and assists in promoting their fusion with cell membranes, which is crucial for the efficient release of the encapsulated mRNA into the cytoplasm.
3. Phospholipids contribute to the structural integrity of the LNPs, mimicking the natural components of biological membranes, which aids in cellular uptake.
4. PEG is incorporated to increase the stability and circulation time of the nanoparticles by reducing their recognition and clearance by the immune system. However, PEGylated lipids can sometimes induce allergic reactions in certain individuals.

There are different types of LNPs based on their formulation and specific applications. These include cationic liposomes, neutral liposomes, and solid lipid nanoparticles (SLNs), each offering distinct advantages for mRNA delivery. For example, cationic liposomes tend to form more stable complexes with mRNA, but they may also induce more immunogenic responses. Neutral liposomes, on the other hand, are less likely to provoke immune reactions, making them suitable for certain therapeutic applications. Solid lipid nanoparticles, another class of LNPs, offer better stability and control over release kinetics, making them useful in specific drug delivery scenarios.

**Figure 2:**
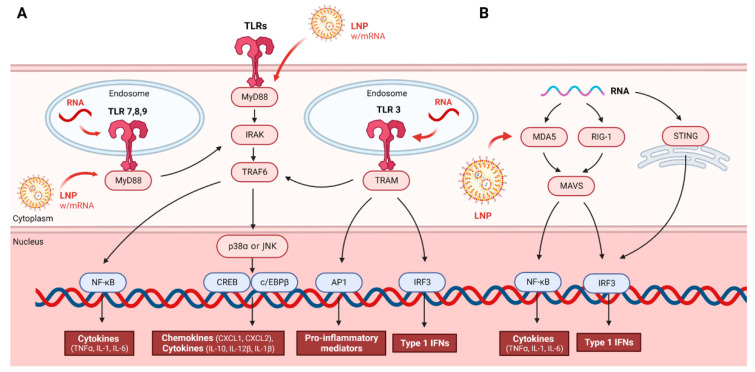
TLR and RLR signaling pathways and LNP effects. (**A**) Toll-like receptors (TLRs) include TLRs 3, 7, 8, and 9, which are located on the endosomal membrane, while other TLRs reside on the cell surface. Upon recognizing foreign antigens, TLRs initiate downstream signaling cascades, typically through the MyD88 adapter protein, except in the case of TLR3. This leads to the activation of transcription factors such as AP-1, NF-κB, CREB, c/EBP, and IRF3, which translocate to the nucleus to induce an innate immune response. The activation of these transcription factors stimulates the production of cytokines, chemokines, and type 1 interferons (IFNs). Endosomal TLRs 3, 7, 8, and 9 recognize RNA, and mRNA encapsulated in lipid nanoparticles (LNPs) can engage the MyD88 signaling pathway. (**B**) The retinoic acid-inducible gene I-like receptor (RLR) pathway is triggered when cytosolic RNA helicases, such as MDA5 and RIG-I, detect foreign RNA, or when STING proteins on the endoplasmic reticulum are activated. This pathway signals through the mitochondrial antiviral signaling protein (MAVS), leading to the activation of transcription factors NF-κB and IRF3, which induce cytokines and type 1 IFNs. LNPs can activate MDA5, enhancing the immune response by stimulating these pathways. Reprinted with permission from Ref. [36] under the Creative Commons Attribution 4.0 International License.

LNPs facilitate the delivery of mRNA into cells by fusing with the cell membrane, allowing the RNA to enter the cytoplasm, where it can be translated into the target protein. The key components of LNPs include ionizable lipids, cholesterol, phospholipids, and polyethylene glycol (PEG). Ionizable lipids are essential for enabling the LNP to interact with the negatively charged RNA, while cholesterol provides stability and helps promote the fusion of LNPs with cell membranes [33]. LNPs are not only protective but also enhance the immune response generated by mRNA vaccines. For example, they can act as adjuvants, substances that boost the immune response to the vaccine. This was crucial in the development of COVID-19 vaccines, as a strong immune response was needed to ensure protection against the rapidly spreading virus [6]. However, LNPs are not without limitations. Some individuals may experience allergic reactions to PEGylated lipids, and LNPs tend to accumulate in the liver, which may lead to off-target effects or toxicity [34]. Despite these limitations, LNPs have proven to be the most effective and scalable delivery system for mRNA vaccines to date. The lessons learned from their use in COVID-19 vaccines are likely to inform the development of future RNA-based therapies, including mRNA vaccines for other infectious diseases, cancer immunotherapies, and treatments for rare genetic disorders. Moreover, ongoing research is focused on refining LNP technology to reduce immunogenicity, enhance tissue-specific targeting, and improve the overall safety and efficacy of RNA delivery [35]. Figure 2 illustrates the crucial roles of TLR and RLR signaling pathways in recognizing foreign RNA and initiating the innate immune response. TLRs located on both the endosomal and cell surface engage signaling pathways, particularly through MyD88 (except for TLR3), leading to the activation of key transcription factors like NF-κB and IRF3 [36]. This results in the production of cytokines and type 1 IFNs, which are essential for antiviral defense. The RLR pathway, triggered by cytosolic sensors MDA5 and RIG-I, also leads to immune activation via MAVS. Lipid nanoparticles (LNPs) encapsulating mRNA can interact with these pathways, enhancing immune responses, particularly by activating TLRs and RLRs, thus demonstrating their potential in immunotherapies and vaccines.

### 3.3. Regulatory Acceleration and Emergency Use Authorizations

The COVID-19 pandemic also triggered a major shift in the regulatory landscape for RNA therapeutics, particularly through the issuance of Emergency Use Authorizations (EUAs). Regulatory agencies worldwide, including the FDA, EMA, and others, had to adapt rapidly to the pressing need for effective vaccines. Traditionally, the regulatory pathway for new drugs and vaccines is lengthy, involving multiple phases of clinical trials, review periods, and post-marketing surveillance. However, the urgent need to curb the pandemic prompted regulators to implement accelerated pathways for mRNA vaccines [37]. In the case of Pfizer-BioNTech’s BNT162b2 and Moderna’s mRNA-1273, both vaccines received EUAs in December 2020, less than a year after the outbreak of COVID-19. These EUAs allowed for the temporary use of the vaccines while the companies continued to collect data from ongoing clinical trials. This unprecedented speed was made possible by adaptive clinical trial designs, rolling reviews of data by regulatory agencies, and increased collaboration between manufacturers and regulators [38]. One of the key factors in the rapid regulatory approval was the accumulation of extensive preclinical and early-phase clinical data on mRNA vaccines and LNP delivery systems before the pandemic. While mRNA vaccines had not previously been approved for widespread use, their safety profiles were well understood from studies on mRNA-based cancer vaccines and other applications. This pre-existing knowledge allowed regulators to assess the safety and efficacy of mRNA vaccines more quickly than would have been possible for completely novel technologies [39]. The regulatory success of mRNA vaccines during the pandemic has had lasting implications for the future of RNA therapeutics. Many experts believe that the pandemic has demonstrated the feasibility of accelerated regulatory pathways for novel therapies, potentially paving the way for faster approvals of other RNA-based treatments in the future. However, it also highlighted the importance of rigorous post-marketing surveillance to ensure long-term safety and efficacy, especially for technologies that are being deployed on a large scale for the first time [40].

### 3.4. Global Impact and Distribution of mRNA Vaccines

The global impact of mRNA vaccines during the COVID-19 pandemic cannot be overstated. By the end of 2021, billions of doses of mRNA vaccines had been distributed worldwide, helping to curb the spread of the virus and reduce the severity of illness in those who contracted it. The global distribution of these vaccines posed significant logistical challenges, particularly in terms of manufacturing, cold chain requirements, and equitable access [41]. One of the major hurdles in the distribution of mRNA vaccines was their reliance on cold storage. Pfizer-BioNTech’s vaccine initially required storage at ultra-low temperatures of around −70 °C, while Moderna’s vaccine required storage at −20 °C. This created significant logistical challenges, particularly in low- and middle-income countries where cold chain infrastructure is limited. Over time, improvements in storage conditions, such as the development of more temperature-stable formulations, helped alleviate some of these challenges, but cold chain requirements remain a key consideration for future mRNA-based vaccines and therapies [42,43]. In terms of global access, the distribution of mRNA vaccines was initially concentrated in high-income countries that had the resources to secure large quantities of doses. However, initiatives such as the COVAX facility, led by the World Health Organization (WHO), sought to ensure more equitable access to vaccines by facilitating donations and distribution to lower-income nations. The global rollout of mRNA vaccines underscored the need for greater investment in global vaccine manufacturing capacity and infrastructure to ensure that all regions have access to life-saving therapies in future pandemics [44]. Moreover, the success of mRNA vaccines during the COVID-19 pandemic has spurred interest in the development of other mRNA-based vaccines and therapies. Researchers are now investigating mRNA vaccines for a variety of infectious diseases, including influenza, Zika, and HIV. Additionally, the potential of mRNA technology extends beyond infectious diseases, with promising applications in cancer immunotherapy, personalized medicine, and rare genetic disorders [45].

## 4. Lipid Nanoparticles (LNPs) in RNA Delivery

Lipid nanoparticles (LNPs) have been pivotal in the advancement of RNA therapeutics, particularly in the delivery of mRNA vaccines during the COVID-19 pandemic. As RNA molecules are inherently unstable and prone to degradation by nucleases, the development of effective delivery systems has been essential to ensuring their therapeutic potential. LNPs have emerged as the most effective platform for RNA delivery, offering protection from degradation and facilitating cellular uptake. This section discusses the mechanism of action, recent advances in LNP formulation, the comparative efficacy of LNPs versus other delivery systems, and their safety profile and immunogenicity [46,47]. Nanoprimers are an emerging concept in the field of drug delivery and nanomedicine, designed to enhance the efficacy of nanoparticle-based therapies, including mRNA therapeutics. They are small, functional agents that can “prime” target cells or tissues for more efficient uptake of nanoparticles. Nanoprimers work by modifying the local microenvironment, altering cell membrane properties, or interacting with specific receptors on the surface of target cells to facilitate nanoparticle entry. This concept stems from the challenges associated with the delivery of nanoparticles alone, which often struggle with issues such as limited stability, low bioavailability, and insufficient cellular uptake. By incorporating nanoprimers into the delivery system, the interaction between nanoparticles and target cells becomes more efficient, ensuring that the therapeutic payload, such as mRNA, reaches its intended site and exerts its biological function more effectively. Nanoprimers are particularly useful in overcoming biological barriers like the extracellular matrix or immune recognition, which can otherwise hinder nanoparticle delivery. As research in this area progresses, nanoprimers hold great potential in improving the specificity and efficiency of mRNA-based therapies for a wide range of applications, including cancer immunotherapy, genetic disorders, and infectious diseases.

**Figure 3:**
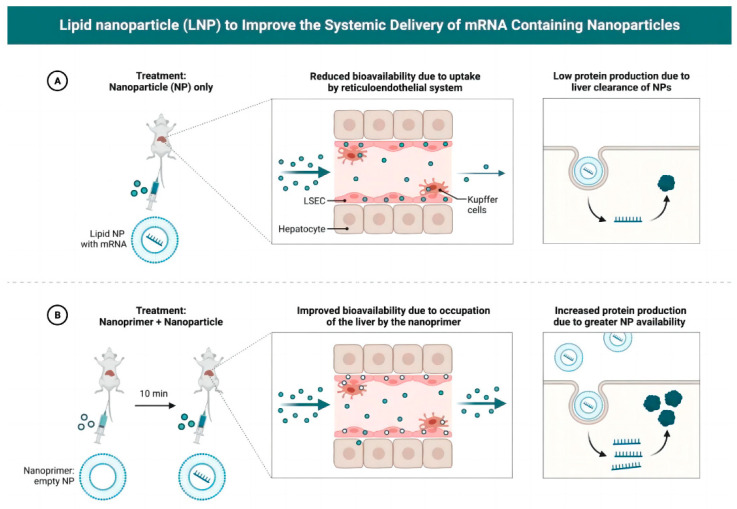
Lipid nanoparticles (LNPs) enhancing the systemic delivery of mRNA-loaded nanoparticles. (**A**) Administration of nanoparticles (NPs) alone. (**B**) Administration of nanoparticles combined with nanoprimers. Reprinted with permission from Ref. [48] under the Creative Commons Attribution 4.0 International License.

Figure 3 demonstrates the critical role of lipid nanoparticles (LNPs) in enhancing the systemic delivery of mRNA-loaded nanoparticles, comparing two different approaches. In panel (A), the challenges of delivering mRNA using nanoparticles alone are highlighted. Although nanoparticles can serve as protective carriers for mRNA, they often face several hurdles, such as limited stability, inefficient cellular uptake, and potential immune system recognition, all of which can reduce their effectiveness. These obstacles can lead to premature degradation of mRNA [48], limiting its therapeutic potential. LNPs improve the delivery by providing a more stable and protective environment, yet further optimization is needed to overcome these inherent limitations in targeting specific tissues and ensuring effective delivery. Panel (B) introduces an innovative strategy where nanoparticles are combined with nanoprimers, which act as a bridge to enhance the targeting and uptake of the nanoparticle–mRNA complexes by cells. Nanoprimers are small, functional entities that can interact with cell surface receptors or modify the microenvironment to promote efficient nanoparticle entry into target cells. This co-delivery system not only improves the stability and protection of the mRNA cargo but also increases its cellular uptake, allowing for more efficient translation of the mRNA into functional proteins. By enhancing both delivery and therapeutic efficacy, the use of nanoprimers addresses the critical challenges of low bioavailability and tissue-specific targeting in mRNA therapeutics. The figure emphasizes the synergy between LNPs and advanced delivery strategies like nanoprimers, highlighting the importance of exploring combined approaches to optimize mRNA therapy. These advancements in delivery mechanisms are essential for improving the clinical application of mRNA therapeutics across a variety of diseases, including cancer, genetic disorders, and viral infections, where precise and efficient mRNA delivery is critical.

### 4.1. Mechanism of Action: How LNPs Enhance RNA Stability and Delivery

LNPs play a critical role in stabilizing RNA molecules and enhancing their delivery into target cells. Naked RNA molecules are vulnerable to rapid degradation by extracellular RNases, enzymes that are abundant in the bloodstream and other bodily fluids. LNPs protect RNA by encapsulating it within lipid bilayers, which shield it from enzymatic degradation and facilitate its circulation in the bloodstream [49]. The core mechanism by which LNPs deliver RNA into cells involves several steps. First, the LNPs, which are typically 50–100 nanometers in size, are taken up by cells through endocytosis, a process where the cell membrane engulfs the nanoparticle. Once inside the cell, the LNPs undergo endosomal escape, a crucial step for successful RNA delivery. Without endosomal escape, the encapsulated RNA would be degraded in lysosomes. LNPs are engineered to release RNA into the cytoplasm by disrupting the endosomal membrane, often through pH-dependent interactions between the ionizable lipids in the LNPs and the acidic environment of the endosome. Once in the cytoplasm, the RNA can be translated into the therapeutic protein or engage with cellular machinery for gene silencing, depending on the type of RNA delivered (mRNA, siRNA, etc.) [50]. The structure of LNPs is composed of four main components: ionizable lipids, cholesterol, helper lipids, and polyethylene glycol (PEG). Ionizable lipids are key for enabling the encapsulation of RNA molecules and promoting their fusion with the cell membrane. Cholesterol and helper lipids contribute to the stability and fluidity of the LNPs, ensuring their structural integrity in biological environments. PEG is added to increase the circulation time of the LNPs by reducing opsonization, the process by which the immune system tags particles for clearance. This composition allows LNPs to remain in circulation long enough to reach target cells and deliver their cargo efficiently [51].

### 4.2. Advances in LNP Formulation and Design

The formulation and design of LNPs have undergone significant advancements in recent years, particularly in response to the need for improved RNA delivery during the COVID-19 pandemic. Early LNP formulations were developed for delivering siRNA, with the first FDA-approved siRNA drug, patisiran, using LNPs to treat hereditary transthyretin amyloidosis. However, the success of mRNA vaccines during the pandemic has accelerated innovation in LNP design, with a focus on enhancing their efficiency, specificity, and safety [52]. One of the major innovations in LNP formulation has been the development of ionizable lipids that are less immunogenic and more effective at promoting endosomal escape. Ionizable lipids are designed to be neutral at physiological pH but acquire a positive charge in the acidic environment of the endosome, which facilitates the disruption of the endosomal membrane and the release of RNA into the cytoplasm.

These advances have improved the efficiency of RNA delivery, reducing the amount of RNA needed for a therapeutic effect and minimizing potential side effects associated with higher doses [53]. Another significant area of advancement is the optimization of PEGylation. While PEG helps increase circulation time, it can also reduce the efficiency of RNA delivery by hindering the fusion of LNPs with cell membranes. Recent research has focused on balancing PEGylation to achieve optimal pharmacokinetics without compromising delivery efficiency. For example, shorter PEG chains or PEG alternatives were explored to maintain circulation time while enhancing cellular uptake [54].

**Figure 4:**
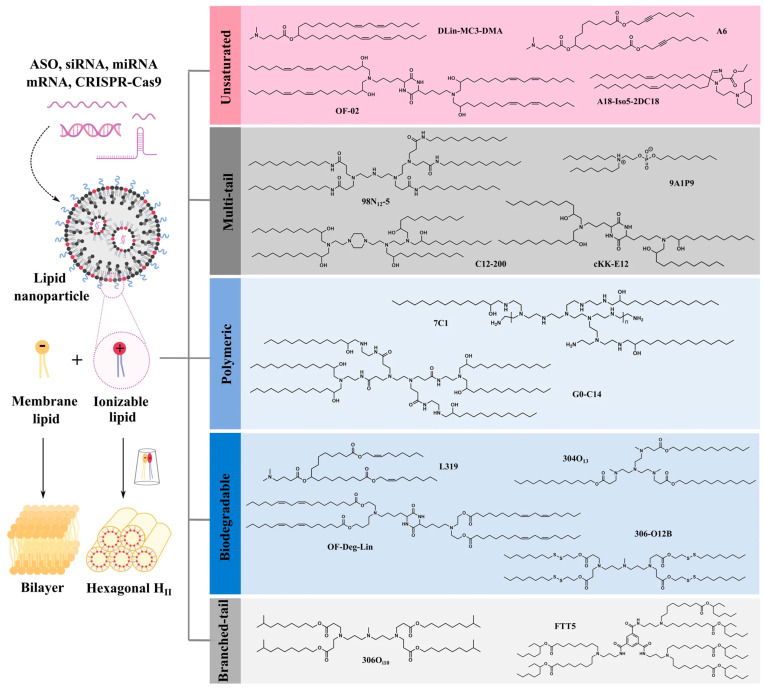
Mechanism of endosomal disruption by ionizable lipids and the five main structural types of ionizable lipids. Ionizable lipids, once protonated within the acidic environment of endosomes, form cone-shaped ion pairs with the anionic phospholipids present in the endosomal membrane. This interaction disrupts the lipid bilayer, facilitating the escape of RNA from the endosome into the cytosol. Ionizable lipids used for RNA delivery can be classified into five distinct structural categories: (1) unsaturated lipids, which contain double bonds; (2) multi-tail lipids, which possess more than two hydrocarbon tails; (3) polymeric lipids, which include polymer or dendrimer structures; (4) biodegradable lipids, which contain bonds that can degrade; and (5) branched-tail lipids, characterized by branched hydrocarbon chains. Reprinted with permission from Ref. [53] under the Creative Commons Attribution 4.0 International License.

Figure 4 highlights the role of ionizable lipids in facilitating RNA release from endosomes, a crucial step in the successful delivery of RNA-based therapeutics. Ionizable lipids are engineered to become protonated in the acidic environment of the endosome, forming cone-like structures by interacting with negatively charged phospholipids within the endosomal membrane. This ion pairing disrupts the membrane’s integrity, creating pathways for the encapsulated RNA to escape into the cytosol, where it can execute its intended therapeutic functions, such as protein translation [53]. The figure also classifies ionizable lipids into five key structural categories—unsaturated, multi-tail, polymeric, biodegradable, and branched-tail lipids. These different structural designs are aimed at optimizing interactions with biological membranes, thus improving RNA release efficiency and promoting successful endosomal escape. For example, unsaturated lipids are more fluid and enhance membrane fusion, while biodegradable lipids can be broken down into less toxic byproducts, minimizing side effects. Additionally, the development of tissue-targeted lipid nanoparticles (LNPs) is an area of ongoing research. Since LNPs naturally accumulate in the liver, which is beneficial for liver-targeted therapies, new strategies are needed to direct LNPs to other tissues like the lungs, spleen, or tumors. This can be achieved by modifying the surface of LNPs with targeting ligands, such as antibodies, peptides, or small molecules, which can selectively bind to receptors on specific cell types. Such advances could significantly broaden the applications of RNA-based therapies, enabling precision medicine approaches for treating cancers, genetic disorders, and other diseases. By combining optimized ionizable lipids for endosomal escape with targeted delivery strategies, the overall efficacy and safety of RNA therapeutics could be significantly improved, paving the way for next-generation treatments.

This strategy is particularly promising for applications in cancer therapy, where delivering RNA to tumor cells while sparing healthy tissues is crucial [55]. Additionally, advances in high-throughput screening and computational modeling have facilitated the rapid design and testing of new LNP formulations. These tools allow researchers to systematically evaluate the effects of different lipid components on the efficiency, safety, and stability of RNA delivery systems, accelerating the discovery of next-generation LNPs [56]. Also, exosomes are naturally occurring extracellular vesicles that can transport RNA, proteins, and lipids between cells. They have garnered interest as a potential delivery system for RNA therapeutics due to their biocompatibility and ability to evade immune detection. However, the use of exosomes for RNA delivery is still in its early stages, and challenges remain in scaling up production, achieving consistent loading of RNA cargo, and ensuring targeted delivery. LNPs, by contrast, are more readily scalable for mass production and have been extensively tested in clinical settings, giving them a clear advantage in terms of clinical translation [57].

Targetability is a crucial area of research aimed at improving the specificity and efficacy of RNA-based therapeutics. While lipid nanoparticles (LNPs) naturally accumulate in the liver, which is advantageous for treating liver diseases, the ability to target other tissues or organs remains a significant challenge. Current efforts are focused on modifying LNP surfaces with targeting ligands, such as antibodies, peptides, or aptamers, to direct them to specific cell types. These ligands bind to receptors that are overexpressed on target cells, such as cancer cells or specific immune cells, allowing for more precise delivery of RNA payloads. For example, targeting ligands can be used to direct RNA therapeutics to tumors, enhancing the efficacy of cancer vaccines or RNA interference (RNAi) treatments. Additionally, organ-specific delivery, such as to the lungs or spleen, is being explored for diseases like cystic fibrosis or certain immune disorders. Improving targetability is key to expanding the therapeutic potential of RNA-based treatments while reducing off-target effects and toxicity.

### 4.3. Comparative Efficacy: LNPs vs. Other Delivery Systems

While LNPs are currently the most widely used delivery system for RNA therapeutics, several alternative delivery platforms have been explored, each with its own strengths and limitations. Comparing the efficacy of LNPs to other systems, such as polymeric nanoparticles, exosomes, and viral vectors, highlights the advantages and challenges of LNPs in RNA delivery.

Polymeric Nanoparticles: Polymeric nanoparticles are an alternative to LNPs that have been used for delivering RNA and other nucleic acids. These nanoparticles are composed of biodegradable polymers, such as poly(lactic-co-glycolic acid) (PLGA) or polyethylenimine (PEI), which protect the RNA from degradation and facilitate its uptake by cells. While polymeric nanoparticles offer stability and biocompatibility, they often struggle with efficient endosomal escape, which limits their ability to deliver RNA into the cytoplasm. LNPs, in contrast, have a more efficient mechanism for endosomal escape, making them more effective at delivering RNA into cells [58].

Viral Vectors: Viral vectors, such as adenoviruses and lentiviruses, have been widely used for gene therapy and RNA delivery. They offer high transduction efficiency, meaning they can effectively deliver RNA into a wide range of cell types. However, viral vectors can elicit strong immune responses, which limits their repeated use in patients. LNPs, on the other hand, are less immunogenic and can be administered multiple times with fewer adverse immune reactions, making them more suitable for chronic or repeated treatments [59].

Overall, LNPs offer a unique combination of biocompatibility, scalability, and efficiency in delivering RNA into cells, which has made them the preferred delivery system for mRNA vaccines and other RNA-based therapies. While alternative delivery platforms hold promise, LNPs currently lead in terms of clinical success and widespread application [60]. Table 1 provides a comparative analysis of various RNA delivery systems, including lipid nanoparticles (LNPs), polymeric nanoparticles, and viral vectors. It highlights the key advantages and disadvantages of each platform, emphasizing their distinct strengths and limitations in RNA-based therapeutic applications.

### 4.4. Safety Profile and Immunogenicity of LNPs

While LNPs have demonstrated remarkable efficacy in delivering RNA therapeutics, their safety profile and potential immunogenicity are critical considerations for their continued use in clinical settings. The immunogenicity of LNPs refers to their ability to trigger immune responses, which can be both beneficial and detrimental depending on the context. One of the key safety concerns associated with LNPs is the potential for allergic reactions, particularly to the polyethylene glycol (PEG) component. PEG is commonly used to extend the circulation time of LNPs by preventing their rapid clearance by the immune system [61]. However, some individuals may develop anti-PEG antibodies, leading to hypersensitivity reactions upon repeated administration of PEGylated LNPs [62]. In rare cases, these reactions can result in anaphylaxis, a severe and potentially life-threatening allergic response [63]. To mitigate this risk, researchers are exploring alternative stabilizers that do not elicit immune responses, such as biodegradable polymers or PEG alternatives [64]. Another concern is the potential for LNPs to elicit inflammatory responses. While LNPs used in mRNA vaccines have been shown to generate an immune response against the target antigen (e.g., the SARS-CoV-2 spike protein), they can also stimulate innate immune pathways in a non-specific manner. This can result in the production of pro-inflammatory cytokines, which, in some cases, may contribute to vaccine-related side effects such as fever, fatigue, and injection site pain [65]. However, these side effects are generally mild and transient, and the overall safety profile of LNP-based vaccines has been deemed acceptable by regulatory agencies [66]. Despite these concerns, the immunogenicity of LNPs can be advantageous in certain therapeutic contexts. For example, in cancer immunotherapy, the ability of LNPs to stimulate the immune system can enhance the therapeutic efficacy of RNA-based cancer vaccines by promoting a stronger anti-tumor immune response. The challenge lies in balancing the immunostimulatory effects of LNPs to maximize therapeutic benefit while minimizing adverse reactions [67]. The biodistribution of LNPs is another important factor influencing their safety. LNPs tend to accumulate in the liver due to their uptake by hepatocytes and resident immune cells, such as Kupffer cells. While this liver tropism is beneficial for RNA therapies targeting liver diseases, it may lead to off-target effects or hepatotoxicity in other therapeutic applications. Ongoing research aims to modify the lipid composition of LNPs to reduce liver accumulation and achieve more selective tissue targeting [68]. Therefore, while LNPs have a favorable safety profile, particularly compared to viral vectors, ongoing efforts are needed to further reduce their immunogenicity and improve their tissue-specific delivery. These improvements will be crucial for expanding the therapeutic applications of LNPs beyond mRNA vaccines to include treatments for chronic diseases, genetic disorders, and cancer.

The immunogenicity of lipid nanoparticles (LNPs) is a critical concern in RNA therapeutics, as it can lead to immune responses that may either be beneficial or detrimental. A key issue is the potential for allergic reactions, particularly to polyethylene glycol (PEG), a stabilizer commonly used in LNP formulations. Some individuals develop anti-PEG antibodies, which can trigger hypersensitivity reactions, including rare cases of anaphylaxis. To mitigate these risks, current research is focusing on developing alternative stabilizers, such as biodegradable polymers or non-immunogenic PEG alternatives, to avoid immune responses. Additionally, strategies are being explored to fine-tune the composition of LNPs to reduce pro-inflammatory cytokine production and unwanted immune stimulation. This involves optimizing the lipid structure to balance safety while preserving the beneficial immunostimulatory effects in therapeutic contexts like cancer immunotherapy. Future developments aim to achieve more selective delivery, thereby enhancing efficacy while minimizing adverse reactions.

Ongoing research is focused on addressing the limitations of lipid nanoparticles (LNPs), such as liver accumulation and allergic reactions triggered by the presence of polyethylene glycol (PEG). To overcome the challenge of liver tropism, scientists are exploring modifications to the lipid composition of LNPs to enhance tissue-specific targeting and reduce off-target effects. One approach involves developing alternative lipid formulations that decrease LNP uptake by hepatocytes and immune cells in the liver, thus mitigating the risk of hepatotoxicity in non-liver-targeting therapies. Another area of active research is the exploration of PEG alternatives, such as biodegradable polymers, to reduce immunogenicity. PEGylated LNPs are commonly associated with hypersensitivity reactions, and some individuals develop anti-PEG antibodies that could lead to severe allergic responses, including anaphylaxis. Replacing PEG with less immunogenic or biodegradable alternatives can help minimize these risks while maintaining the stability and prolonged circulation time of the nanoparticles. In the context of cancer treatment, LNPs offer promising potential for delivering mRNA-based therapies, particularly in immunotherapy. Future clinical trials are focusing on using LNPs to deliver mRNA vaccines that target tumor-associated antigens, stimulating a strong anti-tumor immune response. For example, personalized cancer vaccines using mRNA-encoding neoantigens, which are unique to a patient’s tumor, are being developed to enhance the immune system’s ability to recognize and destroy cancer cells. Moreover, LNPs are being evaluated for delivering mRNA-encoding immune checkpoint inhibitors, which could reinvigorate T-cell responses against tumors. These innovations aim to expand the use of LNP-based mRNA technology beyond infectious diseases and into cancer treatment, offering new hope for effective, targeted therapies in oncology.

## 5. Alternative RNA Delivery Systems

While lipid nanoparticles (LNPs) have become the most prominent RNA delivery system, especially with the success of mRNA vaccines during the COVID-19 pandemic, alternative delivery systems are also being explored. These systems aim to improve the efficiency, specificity, and safety of RNA therapeutics. Polymeric nanoparticles, viral vectors, and hybrid delivery systems each offer unique advantages and challenges. In this section, we discuss these alternative RNA delivery platforms and their future potential in advancing RNA therapeutics.

### 5.1. Polymeric Nanoparticles

Polymeric nanoparticles are one of the earliest and most studied alternatives to LNPs for delivering RNA. These particles are composed of biodegradable polymers such as poly(lactic-co-glycolic acid) (PLGA), polyethylenimine (PEI), and chitosan, which encapsulate RNA and protect it from degradation in biological environments. Polymeric nanoparticles offer several advantages, including biodegradability, tunability in terms of size and surface chemistry, and the ability to encapsulate various types of nucleic acids, including mRNA, siRNA, and miRNA [69]. One of the key strengths of polymeric nanoparticles is their versatility. The composition and structure of the polymers can be modified to control the release of RNA, allowing for sustained or targeted delivery. For example, PLGA-based nanoparticles degrade over time in the body, releasing their RNA payload in a controlled manner. This slow release is particularly advantageous for applications that require prolonged gene expression, such as cancer immunotherapy or chronic diseases [70]. Additionally, polymeric nanoparticles can be functionalized with targeting ligands, such as antibodies or peptides, to direct them to specific cell types or tissues [71]. This targeted delivery is essential for treating diseases that require tissue-specific RNA delivery, such as liver or lung diseases. However, polymeric nanoparticles face challenges in achieving efficient endosomal escape, a crucial step for RNA delivery. After being internalized by cells, the nanoparticles are often trapped in endosomes, where the RNA is degraded. To address this issue, researchers have developed pH-sensitive polymers that respond to the acidic environment of the endosome, disrupting the endosomal membrane and releasing the RNA into the cytoplasm [72]. Despite these advancements, polymeric nanoparticles still lag behind LNPs in terms of delivery efficiency, especially for mRNA vaccines, where rapid and efficient translation of mRNA is critical. Another challenge is the potential for polymeric nanoparticles to elicit immune responses or cause toxicity. Some polymers, such as PEI, are known to be cytotoxic at high concentrations due to their strong cationic charge, which can disrupt cellular membranes. As a result, significant efforts have been made to modify polymers to reduce their toxicity while maintaining their RNA delivery capabilities. For instance, PEGylation of polymeric nanoparticles, similar to LNPs, has been used to reduce immune recognition and prolong circulation time in the bloodstream [73]. Overall, polymeric nanoparticles hold promise for RNA delivery, particularly in applications where controlled release and targeted delivery are important. However, improvements in endosomal escape and biocompatibility are necessary for them to rival the efficacy of LNPs in clinical settings.

Delivery systems such as polymeric nanoparticles, dendrimers, and viral vectors represent important innovations in the field of RNA delivery, each offering unique advantages and challenges. Polymeric nanoparticles, composed of biodegradable materials like PLGA or PEI, provide protection for RNA molecules and promote their uptake by cells. However, they often struggle with efficient endosomal escape, a critical step for RNA release into the cytoplasm, limiting their overall delivery efficiency. Dendrimers, with their highly branched, tree-like structure, offer precise control over size and surface chemistry, making them ideal for targeted delivery. Their multivalency enables high RNA payloads and the potential for functionalization with targeting ligands, although toxicity and complex synthesis remain hurdles. Viral vectors, such as adenoviruses and lentiviruses, offer high transduction efficiency and robust RNA delivery but are limited by immunogenicity and safety concerns, especially in repeated use. Overall, these alternative delivery systems hold promise for broadening the applicability of RNA therapeutics, particularly in challenging therapeutic areas such as cancer and genetic disorders, where tissue-specific targeting and long-term efficacy are critical.

### 5.2. Viral Vectors

Viral vectors are another well-established platform for delivering RNA and other nucleic acids. These vectors use modified viruses to deliver RNA into cells, leveraging the natural ability of viruses to infect cells and introduce genetic material. Viral vectors have been widely used in gene therapy and vaccine development, with several FDA-approved gene therapies and viral vector-based vaccines already on the market [74]. Adenoviral vectors, lentiviral vectors, and adeno-associated viral (AAV) vectors are among the most commonly used viral vectors for RNA delivery. Each of these vectors has distinct characteristics that make them suitable for different applications. For example, adenoviral vectors can deliver large DNA or RNA payloads and induce strong immune responses, making them useful for vaccines. Lentiviral vectors, derived from HIV, are capable of integrating their genetic material into the host genome, making them ideal for long-term gene expression in gene therapy [75]. AAV vectors, which are non-integrating, have a lower immunogenicity profile and are often used for gene therapies targeting tissues like the liver or muscle [76]. One of the major advantages of viral vectors is their high transduction efficiency. Viral vectors are highly effective at delivering RNA into cells and achieving robust gene expression, which is why they have been extensively used in gene therapy and cancer immunotherapy. However, their use for RNA therapeutics presents several challenges. First, viral vectors can elicit strong immune responses, particularly adenoviral vectors, which may limit their repeated use in patients. For example, adenoviral vectors used in vaccines, such as the Oxford-AstraZeneca COVID-19 vaccine, have been associated with rare but serious side effects, including thrombotic events [77]. Additionally, viral vectors pose a risk of insertional mutagenesis, where the integration of viral RNA into the host genome may disrupt normal cellular function and lead to unintended consequences, such as cancer. This risk is particularly relevant for lentiviral vectors, which integrate into the host genome. AAV vectors, while safer in this regard, still face challenges in terms of manufacturing, scaling up, and achieving tissue-specific delivery [78]. Despite these challenges, viral vectors remain a powerful tool for RNA delivery, especially in gene therapy applications where high transduction efficiency and long-term gene expression are required. Recent advancements in vector design, such as the development of “gutted” viral vectors that lack viral genes and the use of tissue-specific promoters, have helped reduce immunogenicity and improve the safety of viral vectors [79]. Moreover, research into combining viral vectors with other delivery systems, such as LNPs or polymeric nanoparticles, offers exciting possibilities for overcoming the limitations of current viral vector technologies.

### 5.3. Hybrid Delivery Systems

**Figure 5:**
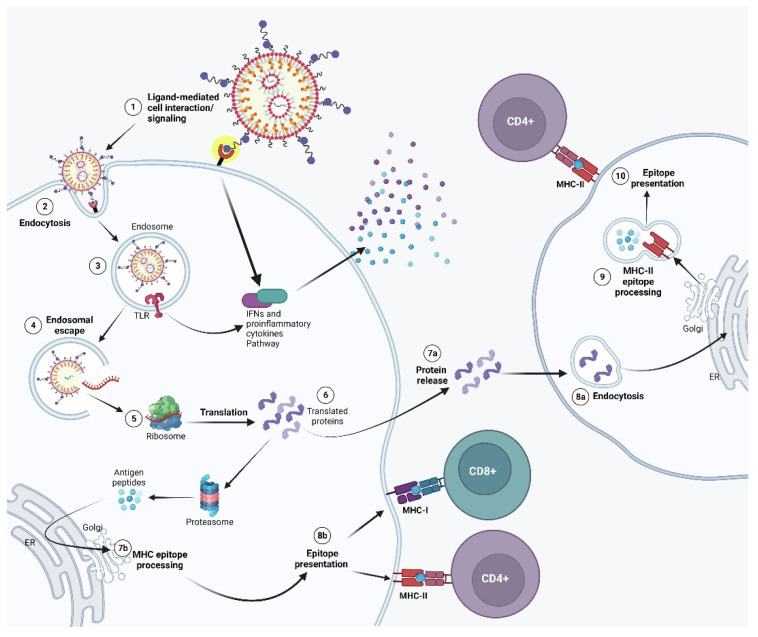
Proposed mechanism of action for APC-targeted LNP–mRNA. The interaction between targeted mRNA–lipid nanoparticles (LNPs) and antigen-presenting cells (APCs) occurs through specific ligands that engage with receptors on the APC surface. This receptor engagement can initiate the production of interferons (IFNs) and other cytokines or chemokines (1). Following this, the LNP–mRNA complex is internalized through endocytosis (2), allowing the mRNA within the endosome to engage with membrane-bound Toll-like receptors (TLRs) (3). The activation of TLRs triggers signaling pathways that lead to the production of Type I IFNs and pro-inflammatory cytokines (4). Subsequently, the mRNA escapes the endosome and is released into the cytosol, where it can be translated by ribosomes (5). The resulting protein may be secreted outside the host cell (7a) and taken up by other APCs (8a), where it is processed into peptides for presentation on MHC class II molecules (9), facilitating recognition by CD4+ T lymphocytes (10). Alternatively, the translated proteins can be degraded into peptides by the proteasome within the same cell (6). These antigenic peptides are transported into the endoplasmic reticulum and can be loaded onto MHC class I and/or class II molecules through a less common pathway (7b). The complexes formed by MHC–peptide epitopes are then presented on the APC surface, where they can bind to the T cell receptor (TCR) of CD8+ and/or CD4+ T lymphocytes (8b). Reprinted with permission from Ref. [83] under the Creative Commons Attribution 4.0 International License.

Hybrid delivery systems combine the strengths of different RNA delivery platforms, such as LNPs, polymeric nanoparticles, and viral vectors, to create more effective and versatile systems. These hybrid systems aim to address the limitations of individual platforms, such as the immunogenicity of viral vectors or the limited endosomal escape of polymeric nanoparticles while maximizing their advantages. One promising approach involves the use of LNP-coated viral vectors, which combine the high transduction efficiency of viral vectors with the protective and delivery-enhancing properties of LNPs. In this system, the viral vector is encapsulated within an LNP, which shields it from immune detection and enhances its circulation time. Once the LNP reaches the target tissue, the viral vector is released, allowing it to transduce cells and deliver RNA efficiently. This hybrid system has the potential to improve the safety and efficacy of viral vector-based RNA therapies, particularly in applications where repeated dosing is necessary [80]. Another hybrid approach involves the use of polymeric nanoparticles in combination with LNPs. Polymeric nanoparticles offer controlled release and tunable delivery properties, while LNPs enhance endosomal escape and protect RNA from degradation. By combining these two systems, researchers can create delivery platforms that offer both sustained release and efficient RNA translation. For example, hybrid nanoparticles that combine PLGA-based polymers with LNPs were shown to improve the delivery of mRNA vaccines and enhance their immune response [81]. Hybrid systems also allow for the incorporation of targeting ligands, such as antibodies, peptides, or aptamers, to direct RNA delivery to specific cell types or tissues. For example, LNP–polymer hybrids were functionalized with antibodies that target cancer cells, enabling more precise delivery of RNA therapeutics to tumors while minimizing off-target effects [82]. Despite their potential, hybrid delivery systems are still in the early stages of development, and challenges remain in terms of manufacturing, scalability, and regulatory approval. However, the flexibility and versatility of these systems make them an exciting area of research, with the potential to revolutionize RNA therapeutics in the future. Figure 5 illustrates the intricate mechanism by which antigen-presenting cells (APCs) interact with APC-targeted lipid nanoparticles (LNPs) loaded with mRNA, a crucial process for eliciting an effective immune response [83]. The initial step involves the binding of specific ligands from the LNPs to receptors on the APC surface, which activates signaling pathways that promote the secretion of interferons (IFNs) and other pro-inflammatory cytokines (1). This cytokine production is vital as it creates an immunostimulatory environment that can enhance the overall immune response. Following the receptor activation, the mRNA–LNP complex is internalized through endocytosis (2). Inside the endosome, the mRNA interacts with membrane-bound Toll-like receptors (TLRs) (3). The engagement of TLRs serves as a key step in recognizing the mRNA as a foreign entity, triggering downstream signaling cascades that lead to the production of Type I IFNs and additional cytokines (4). These responses not only contribute to the innate immune activation but also set the stage for the subsequent adaptive immune response. Once released into the cytosol, the mRNA undergoes translation by ribosomes (5), resulting in the synthesis of proteins that are critical for triggering a robust immune response. These proteins can either be secreted into the extracellular space (7a) and subsequently taken up by other APCs (8a) or be degraded within the same cell (6) into peptide fragments. These peptide fragments are then processed and presented on MHC class II molecules, facilitating recognition by CD4+ T lymphocytes (9 and 10). The pathway where proteins are processed into peptides for MHC class I presentation (7b) is equally important, as it enables the activation of CD8+ cytotoxic T lymphocytes. This dual pathway of MHC presentation underscores the versatility of LNP–mRNA formulations in stimulating both arms of the adaptive immune response, potentially leading to a more comprehensive and effective immunological memory. Therefore, the illustration encapsulates the significance of targeted LNP–mRNA delivery systems in enhancing the activation of immune cells, thereby providing a promising platform for developing vaccines and immunotherapies against various diseases, including cancers and infectious agents.

### 5.4. Extracellular Vesicles Delivery Systems

Extracellular vesicles (EVs), naturally occurring lipid-based carriers, have emerged as a promising alternative to synthetic lipid nanoparticles (LNPs) for RNA delivery, particularly highlighted during the surge of RNA-based therapeutics like mRNA vaccines during the COVID-19 pandemic. EVs share structural similarities with liposomes, classifying them within the broader category of lipid-based delivery systems due to their phospholipid bilayer that enables encapsulation of therapeutic cargo such as RNA. However, unlike synthetic LNPs, EVs are derived from biological sources, making them non-synthetic entities with intrinsic properties that enhance their therapeutic potential. Their endogenous origin offers a lower immunogenic profile, reducing the likelihood of triggering adverse immune responses, a notable advantage in repeated dosing scenarios. Furthermore, EVs possess natural targeting abilities, often retaining surface proteins from their parent cells, which aid in the delivery of cargo to specific tissues or cell types. This capability offers a potential edge over LNPs, which often require external targeting ligands for specificity. With the impact of COVID-19 catalyzing advances in RNA therapeutics, EVs represent a growing area of research, providing a bio-inspired alternative to synthetic delivery platforms, potentially expanding the range and efficacy of RNA-based treatments beyond vaccines to applications like cancer and genetic disorders.

### 5.5. Future Directions in RNA Delivery Technologies

The field of RNA delivery is rapidly evolving, with new technologies and innovations emerging to address the limitations of current systems. As RNA therapeutics expand beyond vaccines into areas such as gene therapy, cancer immunotherapy, and rare genetic disorders, the need for more efficient, targeted, and safe delivery systems becomes increasingly important. One of the key areas of future research is improving the targeting capabilities of RNA delivery systems. While LNPs and other nanoparticles tend to accumulate in the liver, new strategies are being developed to direct these systems to other tissues, such as the lungs, brain, or tumors. For example, researchers are exploring the use of tissue-specific ligands, such as peptides or antibodies, to target specific cell types. Additionally, nanoparticles that respond to specific environmental cues, such as pH or temperature, are being developed to achieve more precise delivery [84]. Another important direction is the development of non-viral delivery systems that can rival the efficiency of viral vectors. LNPs have proven highly effective for delivering mRNA vaccines, but improvements in their design and formulation are needed to enhance their ability to deliver larger RNA molecules, such as those used in gene therapy. Moreover, advancements in endosomal escape mechanisms, such as the use of ionizable lipids or pH-sensitive polymers, will be crucial for improving the efficiency of non-viral delivery systems [85]. In addition to technical advancements, regulatory considerations will play a significant role in shaping the future of RNA delivery technologies. The rapid development and approval of mRNA vaccines during the COVID-19 pandemic have set a precedent for accelerated regulatory pathways for RNA therapeutics. However, ensuring the long-term safety and efficacy of these therapies will require rigorous clinical trials and post-market surveillance. As RNA therapeutics become more widespread, the establishment of clear regulatory guidelines will be essential for ensuring patient safety and fostering innovation [86]. Finally, the integration of RNA delivery technologies with personalized medicine is an exciting area of future research. RNA therapeutics, particularly mRNA and siRNA, can be tailored to individual patients based on their genetic profile, allowing for more precise and effective treatments. For example, personalized cancer vaccines that target specific mutations in a patient’s tumor are currently being developed using RNA delivery platforms. As advances in genomics and RNA sequencing continue, the potential for personalized RNA therapies will only grow [87]. Therefore, while LNPs have emerged as the dominant RNA delivery system during the COVID-19 pandemic, alternative delivery platforms such as polymeric nanoparticles, viral vectors, and hybrid systems offer promising alternatives. Continued innovation in these areas will be crucial for expanding the therapeutic applications of RNA beyond vaccines and into the broader fields of gene therapy, cancer treatment, and personalized medicine.

## 6. Broadening Applications of RNA Therapeutics

The COVID-19 pandemic was a catalyst for the rapid development and widespread adoption of RNA therapeutics, particularly through mRNA vaccines. However, the potential of RNA therapeutics extends far beyond vaccines. With advances in delivery technologies like lipid nanoparticles (LNPs), polymeric nanoparticles, and viral vectors, RNA-based therapies are being explored in oncology, rare genetic diseases, metabolic disorders, and more. In this section, we will discuss the broadening scope of RNA therapeutics in various fields, focusing on the unique challenges and opportunities in each domain.

**Figure 6:**
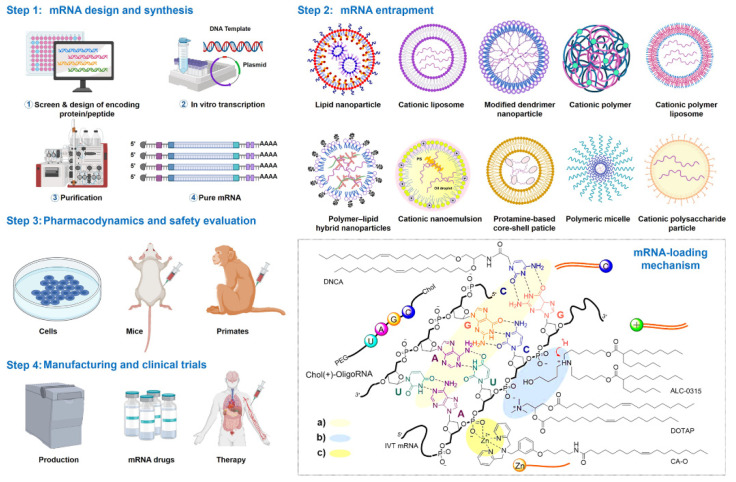
mRNA-based therapeutics: versatile tools in disease treatment. The production process of mRNA-based drugs begins with designing and encoding the target peptide or protein into a plasmid DNA construct. This plasmid DNA is then transcribed into mRNA in vitro using bacteriophage polymerases. The resulting mRNA transcripts are purified through methods such as high-performance liquid chromatography (HPLC) or nanoprecipitation to eliminate impurities and reactants. Purified mRNA is then encapsulated in various delivery systems. The interaction between the mRNA and these vehicles occurs through three mechanisms: (a) electrostatic adsorption involving phosphate groups of ribonucleotides, (b) complementary hydrogen bonding with the nucleotide bases, and (c) coordination with phosphate ions. Delivery systems for mRNA include cationic compounds such as cationic lipids, ionizable lipids, and cationic polymers, along with nucleoside-based lipids like DNCA or amphiphilic polymers such as Chol(+)-oligoRNA. Additionally, metal-based compounds can bind to phosphate ions via coordination. After formulation, the effectiveness, pharmacology, and safety of mRNA drugs are assessed in preclinical models, such as vaccinated mice and primates. Finally, the manufacturing process is scaled up for clinical trials. Reprinted with permission from Ref. [31] under the Creative Commons Attribution 4.0 International License.

Figure 6 illustrates the comprehensive production pipeline for mRNA-based therapeutics, emphasizing their potential as powerful tools for combating diseases. The initial step involves designing the mRNA to encode specific peptides or proteins, which are then inserted into a plasmid DNA construct. This plasmid serves as the template for in vitro transcription, where bacteriophage polymerases synthesize the corresponding mRNA [31]. The purification of mRNA is crucial, as it involves techniques like high-performance liquid chromatography (HPLC) or nanoprecipitation to remove any contaminants, ensuring that the final product is suitable for therapeutic use. Once purified, the mRNA needs to be effectively delivered into target cells, which is achieved by encapsulating it in various vehicles. The interactions between mRNA and these delivery systems can occur through electrostatic adsorption, hydrogen bonding, and coordination with phosphate ions. This versatility in interaction types allows for the use of different delivery vehicles, including cationic lipids, ionizable lipids, cationic polymers, and nucleoside-based amphiphilic polymers. These vehicles are designed to enhance the stability of the mRNA and facilitate its entry into cells, where it can be translated into the desired protein. The figure also highlights the role of metal-based compounds, which can coordinate with the phosphate groups of the mRNA, potentially improving delivery efficiency. Importantly, the efficacy, pharmacological profile, and safety of these mRNA therapeutics are rigorously evaluated in preclinical models, including vaccinated mice and primates, before advancing to clinical trials. This rigorous evaluation is vital to ensure that mRNA therapies are both effective and safe for human use. Overall, the figure underscores the intricate process of developing mRNA-based therapeutics, from design and production to evaluation and clinical application, reinforcing their promise in the field of medicine.

### 6.1. Beyond Vaccines: Therapeutic RNA in Oncology, Rare Diseases, and More

#### 6.1.1. RNA Therapeutics in Oncology

RNA-based therapies have garnered significant interest in oncology due to their ability to modulate gene expression and target cancer cells with high specificity. RNA therapies in cancer primarily involve mRNA-based cancer vaccines, small interfering RNA (siRNA), and antisense oligonucleotides (ASOs). These approaches seek to either stimulate the immune system to recognize and attack cancer cells or directly target oncogenic pathways.

mRNA Cancer Vaccines: Unlike prophylactic vaccines, which prevent disease, mRNA cancer vaccines aim to treat existing malignancies by inducing a robust immune response against tumor-specific antigens. These vaccines work by encoding tumor-associated antigens (TAAs) into mRNA, which is delivered to antigen-presenting cells (APCs). The APCs then present the TAAs to cytotoxic T-cells, stimulating an immune response aimed at destroying tumor cells. The success of mRNA-based COVID-19 vaccines has provided strong proof of concept for cancer vaccines, with multiple mRNA cancer vaccine candidates now in clinical trials [88]. One example is the mRNA vaccine targeting neoantigens, which are unique to a patient’s tumor and not found in healthy tissues. These personalized vaccines are designed based on the genetic profile of a patient’s tumor, and they have shown promise in clinical trials for melanoma and lung cancer [39,89]. However, challenges remain in optimizing the immune response, reducing off-target effects, and scaling up production for personalized therapies.

siRNA and ASOs in Oncology: Small interfering RNA (siRNA) and antisense oligonucleotides (ASOs) are also being explored for cancer therapy due to their ability to silence specific oncogenes. By targeting messenger RNA (mRNA) that encodes for oncogenic proteins, siRNA and ASOs can reduce the expression of these proteins, potentially inhibiting cancer progression. For example, siRNA therapies targeting KRAS mutations—common in pancreatic and colorectal cancers—are currently being developed [90]. However, the delivery of siRNA and ASOs to tumor tissues presents significant challenges. Most RNA molecules, including siRNA, have short half-lives and are susceptible to degradation by nucleases in the bloodstream. Therefore, efficient delivery systems, such as LNPs and polymeric nanoparticles, are critical for ensuring the stability and targeting of these RNA molecules to tumor tissues [91]. Recent advances in nanoparticle design have improved the tissue specificity and safety profiles of these RNA therapies, making them more viable candidates for cancer treatment.

#### 6.1.2. RNA Therapeutics for Rare Genetic Diseases

RNA-based therapies offer a powerful solution for treating rare genetic diseases caused by specific gene mutations. These diseases, which often have no cure, can be addressed by targeting the underlying genetic defect using RNA technologies like siRNA, mRNA replacement therapy, and ASOs.

Spinal Muscular Atrophy (SMA): One of the most successful applications of RNA therapeutics in rare genetic diseases is the treatment of spinal muscular atrophy (SMA), a neurodegenerative disorder caused by mutations in the SMN1 gene. Nusinersen, an ASO therapy, was the first FDA-approved RNA-based treatment for SMA. It works by modifying splicing of the SMN2 gene to increase the production of functional SMN protein, which is lacking in SMA patients [92]. Nusinersen’s success has opened the door for other RNA-based treatments for genetic disorders.

mRNA Replacement Therapy: For genetic diseases where a specific protein is missing or dysfunctional, mRNA replacement therapy can provide a promising treatment option. In this approach, synthetic mRNA encoding the functional protein is delivered to cells, where it is translated into the therapeutic protein. This strategy is being explored for diseases like cystic fibrosis and metabolic disorders. One of the major challenges is delivering the mRNA to the appropriate tissues and achieving sustained protein expression, but advances in LNP and polymeric nanoparticle delivery systems are helping to overcome these obstacles [93].

Gene Silencing for Rare Diseases: In addition to RNA replacement therapies, RNA-based gene silencing techniques such as small interfering RNA (siRNA) are emerging as powerful tools for treating diseases that involve overexpression or toxic gain-of-function mutations. These conditions often result from the production of aberrant or misfolded proteins that cause cellular dysfunction, and traditional therapeutic approaches are limited in their ability to specifically target these disease-causing proteins. siRNA technology, however, directly targets messenger RNA (mRNA) molecules before they can be translated into harmful proteins, thereby offering a precise and efficient means of gene silencing. One of the most notable examples of siRNA’s therapeutic potential is its application in treating transthyretin (TTR) amyloidosis, a rare and progressive disease caused by the accumulation of misfolded transthyretin protein. This accumulation leads to the formation of amyloid fibrils that deposit in tissues such as the heart and peripheral nerves, causing organ dysfunction. TTR amyloidosis can be hereditary, caused by mutations in the TTR gene, or acquired in older individuals. The development of siRNA-based therapies, such as patisiran, represents a significant advancement in the treatment of this condition. Patisiran specifically targets and degrades TTR mRNA in the liver, which is the primary site of TTR protein synthesis. By silencing the expression of the mutated or misfolded TTR protein, patisiran reduces amyloid deposition and alleviates disease symptoms. Patisiran, which was approved by the FDA in 2018, utilizes lipid nanoparticle (LNP) technology for efficient delivery of siRNA to the liver, where it can effectively silence TTR gene expression. LNPs are designed to protect the siRNA from degradation in the bloodstream and facilitate its uptake by liver cells. Once inside the cells, the siRNA is incorporated into the RNA-induced silencing complex (RISC), where it binds to the complementary mRNA sequence of the TTR gene, leading to its cleavage and degradation. This process prevents the production of the misfolded transthyretin protein, addressing the underlying cause of the disease at the molecular level. The approval of patisiran marked a milestone in RNA therapeutics, showcasing the ability of siRNA-based treatments to effectively target and silence specific disease-related genes. Moreover, the success of patisiran has demonstrated the broader potential of siRNA technology for treating a wide range of genetic disorders beyond TTR amyloidosis. Other siRNA-based therapies are currently being developed for conditions such as hypercholesterolemia, hemophilia, and certain types of cancer, where gene silencing can reduce the expression of pathogenic proteins involved in disease progression. The development of siRNA therapeutics also highlights the importance of delivery systems like LNPs, which have played a crucial role in overcoming one of the key challenges of RNA-based therapies: efficient and targeted delivery to specific tissues. LNP technology has enabled the safe and effective delivery of siRNA to hepatocytes, setting the stage for future innovations in delivery systems that could expand the range of treatable diseases by targeting other organs and tissues. Overall, the success of patisiran in treating transthyretin amyloidosis underscores the potential of siRNA-based gene silencing as a transformative approach to tackling rare genetic diseases. As research continues, it is likely that siRNA therapies will be further optimized and expanded to treat other conditions involving toxic protein accumulation or overexpression, offering new hope for patients with currently untreatable diseases [94].

#### 6.1.3. RNA Therapeutics in Cardiovascular Diseases

The application of RNA-based therapies is expanding into the treatment of cardiovascular diseases, which are among the leading causes of morbidity and mortality worldwide. RNA-based therapies offer a novel approach for targeting the molecular mechanisms underlying these diseases, particularly through gene silencing and gene replacement strategies.

siRNA for Cholesterol Management: One of the most promising developments in this field is the use of siRNA to lower cholesterol levels in patients with hypercholesterolemia. Inclisiran, an siRNA therapy targeting proprotein convertase subtilisin/kexin type 9 (PCSK9), has shown significant promise in reducing LDL cholesterol levels. By inhibiting PCSK9, inclisiran allows for increased recycling of LDL receptors, which helps clear LDL cholesterol from the bloodstream [95]. Inclisiran is delivered using LNPs, which protect the siRNA from degradation and facilitate its uptake by liver cells, where PCSK9 is primarily expressed. This therapy is being hailed as a potential game-changer in the management of cardiovascular risk in patients with hypercholesterolemia.

mRNA for Cardiovascular Repair: In addition to gene silencing, mRNA therapies are being explored for regenerative medicine in cardiovascular diseases. One area of research is the use of mRNA to promote the regeneration of damaged heart tissue after myocardial infarction (heart attack). By delivering mRNA that encodes for growth factors or other regenerative proteins, researchers hope to stimulate the repair and regeneration of heart muscle cells, potentially improving outcomes in patients with heart failure [96]. While still in early stages, this approach has the potential to revolutionize the treatment of cardiovascular disease by providing a non-invasive method for tissue repair.

#### 6.1.4. RNA Therapeutics in Infectious Diseases Beyond COVID-19

The success of mRNA vaccines against COVID-19 has spurred interest in developing RNA-based vaccines for other infectious diseases, including influenza, HIV, and malaria. These diseases have proven difficult to combat with traditional vaccine approaches, but RNA-based vaccines offer several advantages, including rapid development, flexibility, and the ability to elicit robust immune responses.

mRNA Vaccines for Influenza: The seasonal flu continues to be a major public health concern, with current vaccines offering limited efficacy due to the constantly evolving nature of the virus. mRNA vaccines are being developed to target more conserved regions of the influenza virus, which may provide broader and longer-lasting protection. Additionally, the rapid adaptability of mRNA vaccine platforms could enable quicker responses to emerging influenza strains, potentially improving vaccine coverage and effectiveness [97].

HIV and Malaria Vaccines: RNA-based vaccines are also being developed for more complex pathogens like HIV and malaria, where traditional vaccines have struggled. In HIV, the high mutation rate of the virus makes it difficult to develop an effective vaccine, but RNA vaccines could be designed to target multiple viral epitopes, increasing the chances of inducing a protective immune response. Similarly, mRNA vaccines for malaria aim to target different stages of the parasite’s life cycle, potentially offering a new strategy for controlling this deadly disease [98].

#### 6.1.5. RNA Therapeutics for Autoimmune and Inflammatory Diseases

RNA-based therapies are being investigated for the treatment of autoimmune and inflammatory diseases, where the goal is often to modulate immune responses rather than replace or silence specific genes. These diseases, such as rheumatoid arthritis, multiple sclerosis, and inflammatory bowel disease, are driven by dysregulated immune pathways that RNA therapies could potentially target.

siRNA and ASOs in Autoimmune Diseases: One approach is to use siRNA or ASOs to silence pro-inflammatory cytokines or other immune mediators that drive disease progression. For example, siRNA therapies targeting tumor necrosis factor-alpha (TNF-α), a key player in rheumatoid arthritis, have shown potential in preclinical studies. By reducing the levels of TNF-α, these therapies could help alleviate the symptoms of autoimmune diseases without the need for systemic immunosuppressants, which can have serious side effects [99].

mRNA for Immune Modulation: In addition to gene silencing, mRNA therapies are being explored for their ability to modulate the immune system. One potential application is the use of mRNA to encode regulatory proteins that promote immune tolerance or reduce inflammation. For instance, mRNA-encoding for interleukin-10 (IL-10), an anti-inflammatory cytokine, could be delivered to immune cells to help control inflammation in diseases like Crohn’s disease or ulcerative colitis [100]. RNA therapeutics have emerged as a transformative field with the potential to revolutionize disease treatment far beyond their initial application in vaccines. The COVID-19 pandemic has significantly accelerated the development and adoption of RNA-based therapies, demonstrating their versatility in addressing both infectious and non-infectious diseases.

Advances in Delivery Technologies: The success of mRNA vaccines has underscored the importance of effective RNA delivery systems, with lipid nanoparticles (LNPs) becoming the preferred method for mRNA delivery due to their stability and efficiency. Ongoing research into alternative delivery systems, including polymeric nanoparticles and viral vectors, continues to enhance targeting and efficacy. Hybrid delivery systems that integrate different technologies are also emerging as promising solutions.

Expanding Therapeutic Applications: RNA-based therapies are now being explored in diverse therapeutic areas. In oncology, mRNA vaccines and RNA interference (RNAi) strategies are being developed to target tumor-specific antigens and oncogenic genes. For rare genetic diseases, therapies such as antisense oligonucleotides (ASOs) and mRNA replacement offer new hope. Additionally, RNA therapeutics are being applied to metabolic, cardiovascular, and autoimmune diseases, showcasing the broad potential of this technology. Despite significant progress, challenges remain in optimizing RNA delivery, minimizing immune responses, and ensuring long-term safety. The integration of RNA technologies into personalized medicine offers exciting possibilities for tailoring treatments to individual genetic profiles. Future research will focus on improving delivery technologies, developing novel RNA-based therapies, and addressing regulatory and manufacturing hurdles. Overall, the impact of COVID-19 on RNA therapeutics has been profound, driving major advancements and expanding the potential applications of RNA-based treatments. As research and development continue, RNA therapeutics are poised to transform medicine by providing innovative solutions for complex and challenging diseases.

### 6.2. Personalized Medicine and RNA-Based Therapies

The advent of RNA-based therapeutics has opened new avenues for personalized medicine, offering tailored approaches to treatment that are designed to address individual genetic profiles and specific disease mechanisms. Personalized medicine aims to optimize therapeutic strategies based on a patient’s unique genetic, environmental, and lifestyle factors. RNA therapeutics, with their ability to directly target and modulate gene expression, have the potential to enhance the precision of medical interventions significantly. This section will explore how RNA-based therapies are being integrated into personalized medicine, discussing the current state of personalized RNA therapies, their potential benefits, and the challenges associated with their implementation.

#### 6.2.1. Tailoring RNA Therapeutics to Genetic Profiles

Genetic and Genomic Advances: The integration of RNA therapeutics into personalized medicine is closely tied to advancements in genomics and genetic sequencing technologies. High-throughput sequencing techniques, such as next-generation sequencing (NGS), allow for comprehensive analysis of an individual’s genome and transcriptome, enabling the identification of genetic variations and expression patterns associated with diseases. These genomic data can be used to design RNA-based therapies that specifically target the genetic alterations responsible for a patient’s condition [17]. For instance, in cancer treatment, genomic profiling of tumors can reveal specific mutations or alterations in oncogenes and tumor suppressor genes. RNA-based therapies, such as mRNA cancer vaccines and RNA interference (RNAi) approaches, can be designed to target these specific mutations. Personalized mRNA vaccines can encode neoantigens that are unique to a patient’s tumor, enhancing the specificity and effectiveness of the immune response against cancer cells [101]. Similarly, RNAi therapies can be developed to silence mutations or overexpressed genes implicated in tumor progression [102].

Personalized RNA Vaccines: The success of mRNA vaccines for COVID-19 has demonstrated the feasibility of personalized RNA vaccines. Personalized cancer vaccines are being developed based on individual tumor profiles, where mRNA sequences are tailored to encode specific tumor-associated antigens. These vaccines aim to elicit a robust immune response against antigens unique to the patient’s cancer, thereby enhancing treatment efficacy [103]. This approach has shown promising results in clinical trials for melanoma and other cancers, where patients received vaccines based on the specific mutations present in their tumors [104].

#### 6.2.2. RNA Therapeutics for Customized Treatments

Customized Treatments for Genetic Disorders: RNA-based therapies offer a particularly powerful tool for treating rare genetic disorders caused by specific genetic mutations. For these diseases, which often have a monogenic basis, RNA-based interventions can be tailored to correct or compensate for the underlying genetic defect. One successful example is the use of antisense oligonucleotides (ASOs) for the treatment of spinal muscular atrophy (SMA). Nusinersen, an ASO therapy, targets the SMN2 gene to increase the production of the SMN protein, which is deficient in SMA patients [105]. Another example is mRNA replacement therapy, where synthetic mRNA is designed to encode the missing or dysfunctional protein in genetic disorders. For instance, mRNA therapies are being developed for cystic fibrosis, where mRNA-encoding the CFTR protein is delivered to the lungs to restore its function [106]. Personalized approaches can further enhance these therapies by tailoring the mRNA sequence to the specific mutation in the patient’s CFTR gene.

RNA-based therapeutics are revolutionizing the treatment landscape for oncology and rare genetic diseases, particularly when integrated with gene editing technologies like CRISPR/Cas9. In oncology, RNA therapies, including mRNA vaccines and small interfering RNAs (siRNAs), have shown promise in enhancing immune responses against tumors or silencing oncogenes. The ability to design mRNA that encodes tumor-associated antigens allows for the generation of personalized cancer vaccines that can train the immune system to target specific cancer cells. Combining mRNA vaccines with CRISPR/Cas9 technology enhances this approach by allowing precise gene editing to introduce or correct mutations in immune cells, potentially increasing their effectiveness against tumors [107].

For rare genetic diseases, the synergy between RNA-based therapies and CRISPR/Cas9 provides a powerful avenue for treatment. In cases where specific genetic mutations are responsible for a disease, RNA molecules can serve as guides for the CRISPR system, enabling precise editing at the desired genomic locations. This capability allows for the direct correction of mutations responsible for conditions such as Duchenne muscular dystrophy or cystic fibrosis, offering a potential cure rather than merely alleviating symptoms. Thus, the integration of RNA technologies with gene editing represents a significant advancement in the development of targeted therapies, paving the way for innovative treatments that address the root causes of diseases.

#### 6.2.3. Customized RNA Therapeutics in Metabolic and Cardiovascular Diseases

Personalized Approaches for Metabolic Disorders: Metabolic disorders, such as phenylketonuria (PKU) and certain types of hyperlipidemia, can also benefit from personalized RNA-based therapies. In PKU, which is caused by mutations in the PAH gene, RNA therapies can be designed to either replace the defective enzyme or reduce the levels of toxic metabolites [108]. Personalized mRNA therapies could be tailored to deliver functional PAH enzymes or to modulate the expression of genes involved in phenylalanine metabolism. For hyperlipidemia, personalized RNAi therapies can target specific genes involved in lipid metabolism. Inclisiran, an siRNA therapy targeting PCSK9, is an example of a personalized approach to managing cholesterol levels [109]. By tailoring the RNAi therapy to an individual’s genetic profile, treatment efficacy and safety can be optimized [110].

Custom mRNA Therapies for Cardiovascular Diseases: In cardiovascular diseases, personalized mRNA therapies can be developed to target specific molecular pathways involved in disease progression. For example, mRNA-encoding for angiogenic factors or anti-inflammatory proteins can be used to promote tissue repair and regeneration after myocardial infarction [111]. By tailoring the mRNA sequences to the patient’s specific condition and response, these therapies can provide more targeted and effective treatments [112]. Custom mRNA therapies represent a promising frontier in treating cardiovascular diseases by targeting specific molecular mechanisms driving disease progression. One approach involves using mRNA to encode proteins that promote angiogenesis, such as vascular endothelial growth factor (VEGF), to enhance blood vessel formation and tissue repair following myocardial infarction. Additionally, mRNA therapies can be designed to produce anti-inflammatory cytokines, which may mitigate the chronic inflammation associated with heart failure and other cardiovascular conditions. The personalized aspect of these therapies allows for the modification of mRNA sequences to address individual patient profiles, optimizing therapeutic efficacy and minimizing potential side effects. Such precision medicine holds great potential for improving outcomes in cardiovascular disease by not only repairing damaged tissues but also modifying the underlying pathophysiological processes contributing to disease progression. Ongoing research into delivery systems and the optimization of mRNA stability will further enhance the feasibility and success of these treatments.

#### 6.2.4. Challenges and Considerations in Personalized RNA Therapeutics

Delivery and Stability: One of the major challenges in personalized RNA therapeutics is the efficient delivery of RNA molecules to the target tissues. RNA molecules are inherently unstable and prone to degradation by nucleases, making effective delivery systems crucial. Lipid nanoparticles (LNPs) have emerged as a leading delivery platform, but their formulation and optimization for personalized RNA therapies remain complex [113]. Customized delivery systems may be needed to address the specific requirements of individual patients and diseases.

Immunogenicity and Safety: Personalized RNA therapies must also address potential issues of immunogenicity and safety. RNA molecules can trigger immune responses, which may lead to adverse effects or reduced therapeutic efficacy. Strategies to minimize immunogenicity, such as optimizing RNA sequences and delivery systems, are essential for ensuring the safety and effectiveness of personalized therapies [114]. Rigorous preclinical and clinical testing is necessary to assess the safety profile of these treatments.

Regulatory and Ethical Considerations: The development and implementation of personalized RNA therapies also involve regulatory and ethical considerations. Personalized therapies may face additional regulatory scrutiny due to their individualized nature, requiring comprehensive clinical data to support their safety and efficacy. Additionally, ethical issues related to genetic privacy and the use of genetic information for personalized medicine must be addressed to ensure equitable access and protection of patient rights [115]. The integration of RNA therapeutics into personalized medicine represents a significant advancement in the treatment of a wide range of diseases. By tailoring RNA-based therapies to individual genetic profiles and specific disease mechanisms, personalized medicine has the potential to enhance treatment efficacy and reduce adverse effects. Advances in genomic sequencing, RNA delivery technologies, and combined RNA-based and gene editing approaches are driving the development of personalized RNA therapies. Despite the promising potential, several challenges remain, including optimizing delivery systems, minimizing immunogenicity, and addressing regulatory and ethical concerns. Continued research and innovation in these areas will be crucial for realizing the full potential of personalized RNA therapeutics and improving patient outcomes across various therapeutic domains.

### 6.3. Potential for mRNA in Preventative Medicine

The success of mRNA vaccines in combating COVID-19 has highlighted the transformative potential of mRNA technology not just for therapeutic interventions but also for preventive medicine. Preventive medicine focuses on the proactive management of health to prevent disease before it occurs, and mRNA technology offers innovative approaches to disease prevention across various domains. This section explores the potential of mRNA in preventive medicine, including its application in infectious disease prevention, cancer prophylaxis, and other areas of health management.

#### 6.3.1. mRNA Vaccines for Infectious Disease Prevention

Efficacy of mRNA Vaccines: The rapid development and deployment of mRNA vaccines for COVID-19 have demonstrated their efficacy in preventing infectious diseases. The mRNA vaccines developed by Pfizer-BioNTech and Moderna have shown high levels of efficacy in preventing symptomatic COVID-19 and severe outcomes, such as hospitalization and death [116,117]. The success of these vaccines underscores the potential of mRNA technology to address a broad range of infectious diseases.

Expanding the Scope to Other Infectious Diseases: Following the success of COVID-19 mRNA vaccines, there is considerable interest in applying mRNA technology to other infectious diseases. Researchers are exploring mRNA vaccines for diseases such as influenza, respiratory syncytial virus (RSV), and even more challenging pathogens like HIV and tuberculosis [118,119]. For instance, mRNA vaccines for influenza are being developed to provide broad protection against multiple strains of the virus, potentially reducing the need for annual vaccine updates [120].

Benefits and Challenges: The benefits of mRNA vaccines extend beyond efficacy. They offer rapid development times, the ability to quickly update vaccine components in response to emerging variants, and the potential for broad-spectrum immunity. However, challenges remain, including the need for ongoing research to optimize mRNA stability, delivery, and long-term safety. Additionally, ensuring equitable access to mRNA vaccines globally is crucial for maximizing their preventive impact [121].

#### 6.3.2. mRNA in Cancer Prophylaxis

Personalized Cancer Vaccines: mRNA technology holds significant promise for cancer prevention through the development of personalized cancer vaccines. These vaccines are designed to elicit an immune response against specific tumor-associated antigens or neoantigens unique to an individual’s cancer profile. The approach involves sequencing a patient’s tumor to identify relevant antigens and then developing mRNA vaccines to target those antigens [122]. Personalized cancer vaccines have shown promise in preclinical studies and early-phase clinical trials, offering the potential to prevent cancer recurrence or development in high-risk individuals [123].

Cancer Vaccine Platforms: Beyond personalized vaccines, mRNA platforms are being explored for developing prophylactic vaccines against common cancers. For example, vaccines targeting human papillomavirus (HPV) have already proven effective in preventing cervical cancer and other HPV-related malignancies. mRNA-based vaccines could offer improved options for HPV vaccination and potentially extend protection to other cancers associated with viral infections [124]. While mRNA cancer vaccines offer exciting potential, several challenges need to be addressed. These include the identification of appropriate tumor antigens, optimizing vaccine formulations, and understanding the long-term efficacy and safety of these vaccines. Continued research and clinical trials will be essential to realize the full potential of mRNA-based cancer prevention [125].

#### 6.3.3. mRNA for Other Preventive Health Measures

Cardiovascular Disease Prevention: mRNA technology is also being explored for cardiovascular disease prevention. mRNA-based vaccines and therapies could target risk factors such as elevated cholesterol levels. For instance, mRNA vaccines could be developed to promote the production of protective proteins or enzymes involved in lipid metabolism, offering a new approach to managing cholesterol and reducing cardiovascular risk [126]. Research in this area is still in its early stages but holds promise for future preventive strategies.

Autoimmune Diseases and Other Conditions: mRNA therapeutics have potential applications in preventing autoimmune diseases and other chronic conditions. For example, mRNA vaccines could be designed to modulate immune responses or induce tolerance to specific autoantigens, potentially preventing the onset of autoimmune disorders. Additionally, mRNA technology could be applied to other areas of health management, such as metabolic disorders or chronic inflammatory conditions [127]. The application of mRNA technology in these preventive contexts involves various challenges, including ensuring precise targeting, managing potential immune responses, and developing scalable production methods. Additionally, regulatory considerations and long-term safety evaluations will be critical in the development and implementation of these preventive therapies [128].

#### 6.3.4. Integration into Public Health Strategies

Adoption and Implementation: For mRNA-based preventive therapies to have a significant impact, they must be integrated into public health strategies effectively. This includes addressing logistical challenges related to vaccine distribution, storage, and administration, as well as ensuring public acceptance and access to mRNA vaccines that may influence their future development, especially for lower-income countries. Collaboration between researchers, healthcare providers, and policymakers will be essential in facilitating the widespread adoption of mRNA-based preventive measures [129].

Global Health Impact: The global impact of mRNA technology in preventive medicine could be profound. By providing new tools for preventing a wide range of diseases, mRNA therapeutics have the potential to improve health outcomes on a global scale. However, ensuring equitable access and addressing disparities in healthcare will be crucial for realizing these benefits [130]. Therefore, the potential for mRNA in preventive medicine is vast, spanning infectious disease prevention, cancer prophylaxis, and other health management areas. The advancements driven by the COVID-19 pandemic have laid the foundation for exploring these applications further, with ongoing research and development poised to enhance the scope and efficacy of mRNA-based preventive therapies. As the field continues to evolve, mRNA technology has the potential to transform preventive medicine, offering innovative solutions to some of the most pressing health challenges of our time.

## 7. Regulatory and Ethical Considerations

### 7.1. Lessons from the COVID-19 Pandemic for Regulatory Approvals

The COVID-19 pandemic has significantly influenced regulatory practices and frameworks for approving new therapeutics, particularly RNA-based therapies. The unprecedented speed at which mRNA vaccines were developed, tested, and authorized has provided valuable insights and lessons for future regulatory processes. This section explores these lessons in detail, focusing on how the pandemic has reshaped regulatory approaches and the implications for RNA therapeutics and beyond.

#### 7.1.1. Accelerated Approval Processes

Emergency Use Authorizations (EUAs): The COVID-19 pandemic necessitated rapid development and deployment of vaccines to address the global health crisis. In response, regulatory agencies such as the U.S. Food and Drug Administration (FDA) and the European Medicines Agency (EMA) implemented Emergency Use Authorizations (EUAs) to expedite the availability of vaccines and treatments. EUAs allowed for the use of mRNA vaccines under conditions of high urgency and provided a pathway for accelerated approval based on preliminary evidence of safety and efficacy [131,132]. The success of this approach demonstrated that it is possible to balance the need for rapid access to life-saving interventions with rigorous standards for safety and efficacy. This has set a precedent for how regulatory bodies can respond to future public health emergencies and has shown that expedited processes can be effectively managed without compromising safety [133].

Streamlined Regulatory Pathways: The pandemic highlighted the potential for streamlining regulatory pathways. For instance, the FDA’s rolling review process allowed vaccine developers to submit data incrementally as it became available, rather than waiting for all the data to be collected before a review [134]. This approach not only accelerated the approval process but also facilitated ongoing dialogue between developers and regulators. The success of this method suggests that similar strategies could be applied to other areas of drug development, particularly for emerging technologies such as RNA therapeutics [135].

#### 7.1.2. Integration of Real-World Evidence

Use of Real-World Data (RWD): The pandemic underscored the importance of integrating real-world evidence (RWE) into regulatory decision-making. Data from vaccine rollouts in diverse populations provided valuable insights into the effectiveness and safety of mRNA vaccines beyond controlled clinical trials. For example, real-world studies confirmed the high efficacy of mRNA vaccines in preventing severe COVID-19 and informed recommendations for booster doses [136,137]. The incorporation of RWE into regulatory evaluations can enhance understanding of how therapies perform in broader populations and under varied conditions. This approach can be particularly valuable for RNA therapeutics, where initial clinical trials may have limited diversity or follow-up duration. Leveraging RWE can help regulators make more informed decisions and ensure that treatments are effective and safe for diverse patient populations [138].

Adaptive Clinical Trial Designs: Adaptive trial designs, which allow for modifications to the trial protocol based on interim results, were prominently used during the pandemic. These designs enabled quicker adjustments to study parameters, such as dosing regimens and participant populations, based on emerging data [139]. The flexibility provided by adaptive designs can accelerate the development of RNA-based therapies and facilitate more efficient identification of optimal treatment strategies.

#### 7.1.3. Collaboration and Communication

Public–Private Partnerships: The pandemic demonstrated the effectiveness of public–private partnerships in accelerating therapeutic development. Collaborations between governments, pharmaceutical companies, and academic institutions facilitated the rapid development of mRNA vaccines. Initiatives such as Operation Warp Speed in the U.S. provided funding and support to expedite vaccine research and manufacturing [140]. These collaborations highlighted the importance of leveraging resources and expertise across sectors to address urgent health challenges. For RNA therapeutics, continued collaboration between regulatory agencies, industry stakeholders, and research institutions can drive innovation and streamline the development process [141].

Transparency and Communication: Transparency in regulatory processes and clear communication with the public were critical during the pandemic. Agencies provided regular updates on vaccine development, approval processes, and safety monitoring, which helped build public trust and confidence [142]. Ensuring transparency in regulatory decisions and maintaining open lines of communication are essential for fostering trust and ensuring the successful adoption of new therapies.

#### 7.1.4. Ethical Considerations and Equity

Equity in Access and Distribution: The pandemic highlighted disparities in access to vaccines and treatments, both within and between countries. Ensuring equitable access to RNA-based therapies is a significant ethical consideration, particularly as these technologies advance. The global rollout of COVID-19 vaccines revealed challenges in vaccine distribution and highlighted the need for strategies to ensure that underserved populations receive timely access to new therapies [143]. Efforts to address these disparities include initiatives to increase vaccine availability in low- and middle-income countries and mechanisms to support equitable distribution of future RNA-based therapies. Ethical considerations around equity must be integrated into regulatory frameworks and policy decisions to ensure that advancements in RNA therapeutics benefit all segments of the population [144].

Informed Consent and Safety Monitoring: The pandemic emphasized the importance of informed consent and ongoing safety monitoring for new therapies. Participants in clinical trials and recipients of new vaccines must be fully informed about potential risks and benefits. Additionally, robust systems for monitoring adverse events and ensuring long-term safety are crucial for maintaining public confidence in new treatments [145]. Regulatory agencies have strengthened safety monitoring systems in response to the pandemic, including enhanced pharmacovigilance and post-marketing surveillance. These practices are essential for identifying and addressing any safety concerns that arise after the widespread use of RNA therapeutics.

#### 7.1.5. Future Implications for RNA Therapeutics

Regulatory Precedents for RNA Therapeutics: The regulatory precedents set during the COVID-19 pandemic will influence the approval and development of RNA therapeutics moving forward. The expedited approval processes, integration of real-world evidence, and emphasis on collaboration will likely shape the regulatory landscape for RNA-based therapies. These precedents will facilitate the development of new RNA therapies for a range of conditions, including cancer, genetic disorders, and infectious diseases [146].

Ongoing Research and Adaptation: As RNA therapeutics continue to evolve, ongoing research and adaptation of regulatory frameworks will be necessary. This includes addressing new challenges related to RNA stability, delivery, and personalized medicine. Regulatory agencies will need to remain flexible and responsive to advancements in technology while maintaining rigorous standards for safety and efficacy [147].

Global Collaboration and Standards: The global nature of the COVID-19 pandemic underscored the need for international collaboration and harmonization of regulatory standards. As RNA therapeutics advance, global collaboration will be essential for establishing consistent regulatory practices and facilitating access to innovative treatments worldwide [148]. The COVID-19 pandemic has provided valuable lessons for regulatory approvals, particularly in the context of RNA therapeutics. The experience has demonstrated the feasibility of accelerated approval processes, the integration of real-world evidence, and the importance of collaboration and transparency. These lessons will shape the future of RNA-based therapies and ensure that they are developed and delivered in a manner that prioritizes safety, efficacy, and equity.

### 7.2. Ethical Challenges in Rapid Development and Distribution

The COVID-19 pandemic has presented an unprecedented scenario for the rapid development and distribution of therapeutics, particularly RNA-based therapies such as mRNA vaccines. While the urgency of the situation necessitated swift action, it also brought several ethical challenges to the forefront. These challenges span issues related to informed consent, equity in distribution, the integrity of clinical trials, and the long-term impacts of accelerated therapeutic development. This section explores these ethical challenges in detail, emphasizing their implications for the field of RNA therapeutics and beyond.

#### 7.2.1. Informed Consent and Participant Safety

Informed Consent in Accelerated Trials: Informed consent is a fundamental ethical requirement in clinical research, ensuring that participants are fully aware of the risks and benefits, and understand the nature of the study that they are joining. The rapid pace of COVID-19 vaccine development put significant pressure on maintaining rigorous informed consent processes. While the urgency of the situation justified expedited procedures, there was a need to ensure that participants still received comprehensive information about the trials [149]. In some cases, the speed at which trials were conducted may have limited the time available for thorough discussions with participants. This raised concerns about whether participants were sufficiently informed about potential risks, including the possibility of unknown long-term side effects. Ethical guidelines and regulatory frameworks had to adapt to balance the need for speed with the necessity of maintaining robust informed consent procedures [150].

Safety Monitoring and Risk Management: With accelerated development came the challenge of ensuring participant safety in the face of limited long-term data. The standard protocols for monitoring adverse events and managing risks were stretched to accommodate the rapid pace of vaccine deployment. Enhanced pharmacovigilance systems were established to track adverse events and ensure prompt responses to any safety concerns [151]. However, the ethical challenge of ensuring rigorous safety oversight while expediting the development process remained significant. The rapid development of mRNA vaccines also highlighted the need for transparent communication with participants and the public regarding the evolving nature of safety data. This transparency is crucial for maintaining trust and ensuring that participants and recipients are fully informed about the current understanding of the safety and efficacy of the vaccines [152].

#### 7.2.2. Equity in Distribution and Access

Global Disparities in Vaccine Access: One of the most pressing ethical challenges during the COVID-19 pandemic was ensuring equitable access to vaccines. The rapid development and distribution of mRNA vaccines highlighted stark global disparities in vaccine availability. Wealthier countries were able to secure large quantities of vaccines early on, while low- and middle-income countries faced significant barriers to access [153]. Ethically, it is imperative to address these disparities to ensure that all populations benefit from advancements in therapeutic technologies. Initiatives such as the COVAX facility were established to promote equitable distribution, but challenges in achieving global equity persisted throughout the pandemic [154]. Moving forward, it is essential to develop strategies that ensure fair access to RNA-based therapies, particularly in under-resourced regions.

Distribution Logistics and Prioritization: The distribution of mRNA vaccines also raised ethical questions about prioritization. Early vaccine allocation often prioritized healthcare workers, the elderly, and high-risk populations. While this prioritization was justified based on the need to protect the most vulnerable, it also necessitated difficult ethical decisions about how to balance competing needs and allocate limited resources effectively [155]. Establishing fair and transparent criteria for vaccine distribution is crucial to addressing these ethical challenges. Ensuring that these criteria are based on principles of equity, justice, and public health need can help guide ethical decision-making in the allocation of RNA-based therapies [156].

#### 7.2.3. Integrity of Clinical Trials

Accelerated Trial Timelines and Data Integrity: The accelerated timelines for clinical trials during the COVID-19 pandemic introduced ethical challenges related to the integrity of data and the rigor of trial protocols. While the rapid development of mRNA vaccines was a remarkable achievement, it required adaptations to traditional trial designs and timelines [157]. Maintaining scientific rigor and data integrity under accelerated conditions was essential for ensuring that the vaccines were safe and effective. Ethical concerns arose about the potential for compromised data quality or the omission of critical study phases. Ensuring that trial designs remained robust and that data were analyzed thoroughly was crucial for upholding the integrity of the research [158].

Balancing Speed and Scientific Rigor: The challenge of balancing speed with scientific rigor was a central ethical issue. While the urgency of the pandemic justified expedited processes, it was essential to ensure that these processes did not undermine the quality of the evidence supporting vaccine safety and efficacy. Regulatory agencies and researchers had to navigate the ethical dilemma of maintaining rigorous scientific standards while responding swiftly to the public health crisis [159].

#### 7.2.4. Ethical Implications for Future RNA Therapeutics

Ethical Frameworks for Future Developments: The ethical challenges encountered during the COVID-19 pandemic provide valuable lessons for the development and distribution of future RNA-based therapeutics. Establishing robust ethical frameworks that address issues of informed consent, equity, trial integrity, and long-term safety will be crucial for guiding the responsible development and deployment of RNA therapies [160]. Future regulatory and ethical guidelines should build on the experiences of the pandemic to ensure that RNA-based therapies are developed and distributed in a manner that prioritizes public health, equity, and ethical integrity. Collaborative efforts among regulators, researchers, and ethicists will be essential in shaping these frameworks and addressing emerging ethical challenges.

Global Collaboration and Ethical Standards: Global collaboration in the development of RNA-based therapies must be accompanied by shared ethical standards that promote equity and justice. International cooperation can help harmonize regulatory practices and ensure that ethical considerations are consistently applied across different regions. This collaborative approach is essential for addressing global health challenges and ensuring that advances in RNA therapeutics benefit all populations [161]. Therefore, the rapid development and distribution of RNA therapeutics during the COVID-19 pandemic have highlighted several ethical challenges, including informed consent, equity in access, trial integrity, and long-term safety. Addressing these challenges requires a commitment to ethical principles and ongoing efforts to ensure that new therapies are developed and distributed responsibly. The lessons learned from the pandemic will inform future approaches to RNA therapeutics and contribute to the advancement of ethical standards in biomedical research and public health.

### 7.3. Long-Term Implications for RNA Therapeutics Regulation

The COVID-19 pandemic has dramatically accelerated the development and deployment of RNA-based therapeutics, particularly mRNA vaccines. This rapid progress has not only transformed the landscape of vaccine development but also provided crucial insights into the regulatory frameworks governing RNA therapeutics. As we move beyond the immediate crisis, it is essential to consider the long-term implications for the regulation of RNA therapeutics. This section discusses the key considerations for shaping future regulatory frameworks, informed by the experiences and challenges encountered during the pandemic.

#### 7.3.1. Evolution of Regulatory Frameworks

Adapting Regulatory Pathways: The accelerated development of mRNA vaccines during the COVID-19 pandemic has prompted a re-evaluation of traditional regulatory pathways. The success of Emergency Use Authorizations (EUAs) and rolling reviews demonstrated the feasibility of expediting the approval process without compromising safety and efficacy standards. Moving forward, regulatory agencies may adopt more flexible pathways for RNA therapeutics, incorporating elements of expedited approval while ensuring rigorous evaluation [162,163]. Regulatory frameworks will need to adapt to the unique challenges of RNA therapeutics, such as ensuring stability, optimizing delivery systems, and addressing manufacturing complexities. The experience gained from the pandemic provides a foundation for developing streamlined yet robust regulatory processes tailored to the needs of RNA-based therapies [3].

Harmonization of International Standards: The global nature of the COVID-19 pandemic highlighted the importance of harmonizing regulatory standards across countries. The differences in regulatory requirements and approval processes among nations can create barriers to the global distribution of RNA therapeutics. Future efforts should focus on enhancing international collaboration and standardizing regulatory practices to facilitate the global development and access to RNA-based therapies [164]. Organizations such as the International Council for Harmonisation (ICH) and the World Health Organization (WHO) play critical roles in this harmonization process. By working towards unified standards, these organizations can help ensure that RNA therapeutics are evaluated consistently and effectively across different regions [165].

#### 7.3.2. Enhanced Focus on Safety and Efficacy

Long-Term Safety Monitoring: One of the key lessons from the COVID-19 pandemic is the importance of long-term safety monitoring for RNA therapeutics. While the initial data on safety and efficacy were promising, the long-term effects of mRNA vaccines and other RNA-based therapies are still being studied. Robust post-marketing surveillance systems are essential for detecting and addressing any adverse effects that may emerge over time [166]. Regulatory agencies will need to implement comprehensive safety monitoring protocols, including long-term follow-up studies and pharmacovigilance programs. This approach will help ensure that any potential risks are identified and managed promptly, maintaining public trust in RNA therapeutics [167].

Real-World Evidence (RWE) Integration: The integration of real-world evidence (RWE) into regulatory decision-making has gained prominence during the pandemic. RWE provides valuable insights into how RNA therapeutics perform in diverse populations and under real-world conditions. Incorporating RWE into regulatory evaluations can enhance the understanding of therapeutic effectiveness and safety, leading to more informed decision-making [168]. Future regulatory frameworks should incorporate mechanisms for systematically collecting and analyzing RWE. This includes leveraging data from electronic health records, insurance claims, and patient registries to complement traditional clinical trial data [169]. By integrating RWE, regulators can ensure that RNA-based therapies are continuously evaluated and optimized based on real-world experiences.

#### 7.3.3. Ethical and Social Considerations

Ethical Frameworks for RNA Therapeutics: The ethical challenges encountered during the COVID-19 pandemic underscore the need for robust ethical frameworks for RNA therapeutics. These frameworks should address issues related to informed consent, equity, and long-term safety, ensuring that the development and distribution of RNA-based therapies adhere to ethical principles [170]. Regulatory agencies, ethics committees, and research organizations should collaborate to establish and uphold ethical standards for RNA therapeutics. This includes developing guidelines for conducting ethical research, ensuring transparent communication with the public, and addressing ethical concerns related to the use of RNA technology [171].

Public Trust and Engagement: Maintaining public trust is critical for the successful adoption of RNA therapeutics. The COVID-19 pandemic highlighted the importance of transparent communication and public engagement in building confidence in new therapies. Future regulatory frameworks should include strategies for engaging with the public, addressing concerns, and providing accurate information about RNA-based therapies [172]. Engaging with diverse communities and stakeholders can help ensure that the development and distribution of RNA therapeutics are aligned with societal values and expectations. This collaborative approach can foster trust and promote the responsible use of RNA technology [173]. Therefore, the long-term implications for RNA therapeutics regulation are multifaceted and require careful consideration of evolving regulatory frameworks, safety monitoring, equity, innovation, and ethical standards. The experiences of the COVID-19 pandemic provide valuable insights for shaping future regulatory practices and ensuring that RNA-based therapies are developed and distributed in a manner that prioritizes public health, safety, and equity.

## 8. Challenges and Future Prospects

### 8.1. Addressing Remaining Challenges in RNA Delivery

The rapid advancements in RNA therapeutics, particularly during the COVID-19 pandemic, have showcased the potential of mRNA vaccines and other RNA-based treatments. However, effective delivery of RNA molecules remains a significant challenge. Despite the success of lipid nanoparticles (LNPs) in delivering mRNA vaccines, several issues need to be addressed to enhance the efficacy, safety, and accessibility of RNA therapeutics. This section discusses these challenges and outlines potential solutions.

#### 8.1.1. Stability and Storage

RNA Stability: RNA molecules are inherently unstable and prone to degradation by RNases, which presents a significant challenge for their delivery and storage. The stability of RNA is crucial for ensuring its effectiveness as a therapeutic. During the pandemic, the stability of mRNA vaccines was managed through the use of LNPs, which protect the RNA from degradation and facilitate its delivery into cells [174,175]. Nevertheless, there remains a need for further improvements in RNA stability to reduce reliance on stringent storage conditions and enhance the shelf life of RNA therapeutics. Advances in RNA modification technologies, such as the incorporation of modified nucleotides or the use of encapsulation methods beyond LNPs, could improve RNA stability [176]. Additionally, the development of more robust storage solutions that can maintain RNA stability at higher temperatures could simplify distribution logistics and expand access [177].

Cold Chain Requirements: The cold chain requirements for mRNA vaccines, which often necessitate storage at temperatures as low as −70 °C, pose logistical challenges, especially in low- and middle-income countries [178]. These stringent requirements increase the cost and complexity of vaccine distribution and limit the accessibility of RNA-based therapies. Efforts to address this challenge include the development of RNA formulations that can be stored at higher temperatures. Research into novel delivery systems that do not require ultra-cold storage could significantly impact the feasibility of widespread RNA therapy distribution [179]. Solutions such as lyophilization of RNA formulations or the use of temperature-stable nanoparticle carriers are promising avenues for reducing cold chain dependency [180].

#### 8.1.2. Targeted Delivery and Cellular Uptake

Specificity and Targeting: A major challenge in RNA delivery is achieving specificity and targeting of the RNA to the intended cells or tissues. While LNPs have been effective in delivering mRNA vaccines to target cells, optimizing the delivery system for specific tissues or cell types remains a challenge [181]. Non-specific delivery can lead to off-target effects and reduced therapeutic efficacy. To enhance targeting, researchers are exploring various strategies, including ligand-based targeting, where specific ligands are attached to delivery vehicles to bind to receptors on target cells [182]. Additionally, advances in nanoparticle engineering, such as surface modification with targeting moieties or the use of tissue-specific promoters, are being investigated to improve the specificity of RNA delivery [183].

Endosomal Escape: After cellular uptake, RNA molecules often face challenges in escaping from endosomes, where they can be degraded before reaching their intracellular targets. Improving endosomal escape is crucial for effective RNA delivery [184]. Current strategies to enhance endosomal escape include the use of pH-sensitive polymers or peptides that disrupt endosomal membranes, allowing RNA to reach the cytoplasm [185]. Researchers are also exploring the use of alternative delivery vehicles, such as cell-penetrating peptides or viral vectors, to facilitate endosomal escape and improve the efficiency of RNA delivery [186]. Continued innovation in this area is essential for overcoming the barriers to effective RNA delivery and maximizing therapeutic potential.

#### 8.1.3. Immunogenicity and Safety

Immunogenicity of Delivery Vehicles: The immunogenicity of delivery vehicles, such as LNPs, can impact the safety and efficacy of RNA therapeutics. Immune responses to the delivery system itself can lead to adverse reactions or reduced therapeutic effectiveness [187]. Addressing immunogenicity involves designing delivery vehicles that minimize immune activation while still providing effective RNA delivery. Research into biocompatible and less immunogenic materials for nanoparticle formulation is ongoing. Strategies include using materials that are naturally occurring or modifying the surface properties of nanoparticles to reduce immune recognition [188]. Additionally, optimizing the formulation of RNA delivery systems to minimize the release of pro-inflammatory cytokines can help mitigate potential immunogenic effects [189].

Long-Term Safety: Long-term safety is a critical consideration for RNA therapeutics, especially given the rapid pace of development and deployment. Although mRNA vaccines have shown a favorable safety profile in the short term, the long-term effects of RNA-based therapies need to be thoroughly evaluated [190]. Ongoing post-marketing surveillance and long-term follow-up studies are essential for monitoring safety and identifying any potential late-onset adverse effects. Transparent reporting and continuous monitoring are crucial for maintaining public trust and ensuring that RNA therapeutics remain safe and effective over time [191].

#### 8.1.4. Manufacturing and Scalability

Manufacturing Challenges: The production of RNA therapeutics involves complex and costly processes, including RNA synthesis, purification, and formulation. Scaling up production to meet global demand, particularly in the context of a pandemic, presents significant challenges. The need for high-quality RNA and consistent manufacturing processes is crucial for ensuring the safety and efficacy of RNA-based therapies. RNA synthesis is inherently delicate, requiring in vitro transcription (IVT) with high precision to generate accurate sequences. IVT processes must maintain stringent conditions to prevent contamination and ensure stability, while RNA must undergo rigorous purification to remove any by-products, such as template DNA and enzymes, which could affect therapeutic outcomes. Purification techniques like high-performance liquid chromatography (HPLC) or size-exclusion chromatography are labor-intensive and expensive, adding to the production cost. Furthermore, RNA is highly sensitive to degradation by nucleases, making stable formulation critical. Lipid nanoparticles (LNPs), the most widely used delivery vehicle for mRNA vaccines, must encapsulate RNA effectively while ensuring particle uniformity, a process that remains difficult to scale [192]. The formulation of LNPs requires precise control over the mixing of RNA with lipids, and even small deviations can lead to inconsistent efficacy or unwanted immune responses. Beyond synthesis and formulation, the scale of production required during a global health crisis like COVID-19 introduces additional logistical hurdles. The supply chain for critical raw materials, such as nucleotides and lipids, can be strained, and large-scale facilities require sophisticated equipment for maintaining quality control [193]. Automated purification systems and standardized production methods have helped improve efficiency, but ensuring batch-to-batch consistency remains challenging [194]. As RNA-based therapies evolve, the industry must continue to develop scalable, cost-effective, and standardized manufacturing processes to support the growing demand for vaccines and other RNA therapeutics.

Infrastructure and Capacity Building: Building infrastructure and capacity for RNA therapeutic manufacturing is critical for supporting future development and distribution. Investments in manufacturing facilities, training programs, and supply chain management can enhance the ability to produce RNA therapeutics at scale and address global health needs [195]. Public–private partnerships and collaborations between governments, industry, and research institutions can play a key role in expanding manufacturing capacity and ensuring that RNA-based therapies are accessible to all populations [196].

#### 8.1.5. Regulatory and Approval Processes

Regulatory Challenges: The rapid development and deployment of RNA therapeutics have highlighted the need for adaptive and flexible regulatory processes. Ensuring that regulatory frameworks can accommodate the unique characteristics of RNA-based therapies while maintaining rigorous standards for safety and efficacy is a key challenge [197]. Regulatory agencies are working to streamline approval processes and incorporate lessons learned from the COVID-19 pandemic. This includes developing guidelines for the evaluation of RNA therapeutics, addressing issues related to stability, delivery, and manufacturing, and ensuring that regulatory processes remain responsive to emerging technologies [198].

Harmonization of Regulations: Harmonizing regulatory standards across countries is essential for facilitating the global development and distribution of RNA therapeutics. Differences in regulatory requirements can create barriers to accessing new therapies and complicate the approval process for multinational studies [199]. Efforts to harmonize regulations, through organizations such as the International Council for Harmonisation (ICH) and the World Health Organization (WHO), can help ensure consistent standards and streamline the approval process for RNA therapeutics [200].

### 8.2. Innovations in Delivery Mechanisms

The surge in interest and development of RNA therapeutics during the COVID-19 pandemic has spurred significant innovations in delivery mechanisms. These innovations aim to overcome the challenges associated with RNA delivery and enhance the efficacy and safety of RNA-based therapies. This section explores some of the key innovations in RNA delivery mechanisms and their potential impact on the future of RNA therapeutics.

#### 8.2.1. Advanced Nanoparticle Systems

Targeted Nanoparticles: Advanced nanoparticle systems are at the forefront of innovations in RNA delivery. Targeted nanoparticles, which can specifically bind to receptors on target cells, offer a promising approach to improving the precision and efficacy of RNA delivery [201]. These nanoparticles can be engineered with surface modifications or conjugated with targeting ligands to enhance their specificity for desired cell types or tissues. For example, modifications such as PEGylation (attachment of polyethylene glycol) can increase the circulation time of nanoparticles and reduce nonspecific interactions [202]. Additionally, the use of cell-specific ligands or antibodies on nanoparticles can improve targeting accuracy and minimize off-target effects [203].

Multi-Functional Nanoparticles: Multi-functional nanoparticles that combine multiple therapeutic agents or functionalities in a single delivery system represent another innovative approach. These nanoparticles can be designed to co-deliver RNA therapeutics with other therapeutic agents, such as small molecules or proteins, to enhance treatment efficacy [204]. For instance, multi-functional nanoparticles could deliver mRNA vaccines along with immune modulators to boost the immune response or deliver RNA-based therapies alongside gene editing tools to achieve combined therapeutic effects [205]. Such systems hold promise for addressing complex diseases that require multi-faceted treatment strategies.

#### 8.2.2. Alternative Delivery Vehicles

Viral Vectors: Viral vectors, including adenoviruses, lentiviruses, and vesicular stomatitis viruses, have been explored as alternative delivery vehicles for RNA therapeutics. These vectors can efficiently deliver RNA into cells and offer the potential for high transfection efficiency [206]. However, concerns related to immunogenicity and potential for insertional mutagenesis must be carefully managed. Recent advancements in viral vector technology aim to reduce immunogenicity and enhance safety profiles. This includes the development of viral vectors with modified surface proteins to evade immune detection or the use of self-replicating RNA viruses to improve delivery efficiency [207]. Viral vectors continue to be a valuable tool for RNA delivery, particularly for applications in gene therapy and vaccine development.

Cell-Penetrating Peptides: Cell-penetrating peptides (CPPs) are short peptides that facilitate the delivery of nucleic acids across cell membranes. CPPs can be conjugated with RNA molecules or incorporated into nanoparticles to enhance cellular uptake [208]. Innovations in CPP design focus on optimizing peptide sequences for efficient delivery and minimizing potential toxicity [209]. CPPs offer a versatile approach to RNA delivery and can be used in conjunction with other delivery systems, such as liposomes or nanoparticles, to improve overall delivery efficacy. Research into CPPs continues to explore their potential for delivering a wide range of RNA-based therapies [210].

#### 8.2.3. RNA Modifications and Formulations

Chemical Modifications: Chemical modifications to RNA molecules can enhance their stability, reduce immunogenicity, and improve their delivery. For example, the incorporation of modified nucleotides, such as pseudouridine or 5-methylcytosine, can increase RNA stability and reduce immune activation [211]. These modifications were successfully used in mRNA vaccines to enhance their performance. Ongoing research into RNA modifications aims to further improve the properties of RNA therapeutics. This includes exploring new types of chemical modifications and developing strategies to balance stability with biological activity [212]. Innovations in RNA chemistry are critical for advancing the development of RNA-based therapies.

Advanced Formulations: Innovative formulations, such as lipid–polymer hybrid nanoparticles or dendritic polymers, represent promising approaches for RNA delivery. These formulations combine the advantages of different materials to enhance delivery efficiency and stability [213]. For example, lipid–polymer hybrids can provide better control over the release of RNA and improve cellular uptake compared to traditional lipid nanoparticles [214]. The development of advanced formulations involves optimizing the composition and structure of delivery systems to achieve desired properties. This includes adjusting lipid and polymer ratios, incorporating stabilizing agents, and tailoring the release profiles of RNA therapeutics [215].

#### 8.2.4. Smart Delivery Systems

Responsive Delivery Systems: Smart or responsive delivery systems are designed to release RNA therapeutics in response to specific physiological conditions or stimuli. These systems can include pH-sensitive nanoparticles that release their RNA payload in the acidic environment of endosomes or temperature-sensitive formulations that release RNA at body temperature [216]. Responsive delivery systems offer the potential for precise control over the timing and location of RNA release, improving therapeutic efficacy and reducing side effects. Research in this area focuses on developing responsive materials and optimizing their performance in different biological environments [217].

On-Demand Delivery: On-demand delivery systems, which allow for controlled release of RNA therapeutics at specific times or locations, represent an exciting innovation. These systems can be triggered by external factors, such as light or magnetic fields, to release RNA when needed [218]. On-demand delivery systems offer the potential for more personalized and flexible treatment approaches. For example, they could enable localized delivery of RNA therapeutics to specific tissues or cells, improving treatment outcomes and reducing systemic side effects [219]. Addressing the remaining challenges in RNA delivery and exploring innovations in delivery mechanisms are crucial for advancing the field of RNA therapeutics. Continued research and development in these areas hold the promise of overcoming current limitations and expanding the applications of RNA-based therapies. By addressing issues related to stability, targeting, immunogenicity, and manufacturing, and by embracing innovative delivery technologies, the field of RNA therapeutics can continue to evolve and provide transformative treatments for a wide range of diseases.

### 8.3. Potential for Next-Generation RNA Therapeutics

The COVID-19 pandemic has highlighted the transformative potential of RNA therapeutics, particularly through mRNA vaccines. As we look towards the future, the field of RNA therapeutics is poised for significant advancements. Next-generation RNA therapeutics promise to build upon the successes of current technologies, addressing existing challenges and expanding the scope of RNA-based treatments. This section explores the potential of next-generation RNA therapeutics, including advances in RNA technology, novel therapeutic applications, and emerging delivery systems.

#### 8.3.1. Advances in RNA Technologies

Enhanced RNA Stability and Expression: One of the primary goals for next-generation RNA therapeutics is to enhance the stability and expression of RNA molecules. Current RNA therapies, including mRNA vaccines, have demonstrated the efficacy of RNA delivery, but improving the stability of RNA in biological environments remains a critical challenge [220]. Advances in RNA modification, such as the incorporation of novel nucleotide analogs and chemical modifications, are being explored to enhance RNA stability and reduce immunogenicity [221]. For instance, research into modified nucleotides, such as 5-methylcytosine and pseudouridine, has shown promise in improving the stability and translation efficiency of mRNA [222]. These modifications can also reduce the activation of innate immune responses, leading to safer and more effective RNA therapies. Additionally, novel RNA structures, such as circular RNAs, are being investigated for their potential to offer improved stability and longer half-lives compared to linear RNA [223].

Self-Amplifying RNA: Self-amplifying RNA (saRNA) represents a significant advancement in RNA therapeutics. Unlike conventional RNA, saRNA contains a replicase gene that allows the RNA to replicate itself once inside the cell, leading to increased expression of the therapeutic protein [224]. This approach can potentially reduce the amount of RNA needed for therapeutic efficacy and enhance the duration of therapeutic protein production. The use of saRNA has been explored in vaccine development, where it could offer advantages such as lower doses and prolonged immune responses [225]. Research is ongoing to optimize saRNA systems for various therapeutic applications, including gene therapy and cancer treatment [226].

#### 8.3.2. Novel Therapeutic Applications

Gene Editing and Gene Therapy: RNA-based technologies, including RNA interference (RNAi) and CRISPR/Cas systems, have revolutionized the field of gene editing and gene therapy. Next-generation RNA therapeutics are expected to leverage these technologies to address a broader range of genetic disorders and diseases. RNAi, which involves the use of small interfering RNA (siRNA) or microRNA (miRNA) to silence specific genes, has shown promise in treating diseases caused by gene overexpression or mutation [8]. Advances in RNAi delivery systems and target specificity are likely to expand the therapeutic applications of RNAi-based therapies. Similarly, the combination of CRISPR/Cas systems with RNA-based delivery methods offers the potential for precise gene editing and correction of genetic mutations [227]. Research into improving the efficiency and safety of CRISPR/Cas-based RNA therapies is underway, with the goal of developing treatments for genetic disorders, cancer, and other diseases [228].

Cancer Immunotherapy: RNA therapeutics have the potential to revolutionize cancer treatment through novel approaches such as personalized cancer vaccines and adoptive cell therapies. Personalized cancer vaccines involve the use of RNA to encode tumor-specific antigens, which can stimulate the immune system to target and destroy cancer cells [229]. The success of mRNA vaccines in COVID-19 has paved the way for similar approaches in cancer immunotherapy. Adoptive cell therapies, such as CAR-T cell therapy, can also benefit from RNA-based technologies. For example, RNA-encoding chimeric antigen receptors (CARs) can be used to genetically modify patient-derived T cells to target specific cancer antigens [230]. Innovations in RNA-based cancer immunotherapy hold the promise of more effective and personalized treatments for cancer patients.

#### 8.3.3. Emerging Delivery Systems

Nanoparticle and Lipid-Based Innovations: While lipid nanoparticles (LNPs) have been instrumental in the success of mRNA vaccines, next-generation delivery systems are being developed to address limitations and expand the capabilities of RNA therapeutics. Innovations in nanoparticle design, such as multifunctional nanoparticles or hybrid systems, are being explored to enhance targeting, stability, and cellular uptake [231]. For example, hybrid nanoparticles that combine lipids with polymers or other materials can offer improved delivery characteristics and controlled release profiles [232]. Additionally, the development of nanoparticles with tissue-specific targeting ligands or stimuli-responsive features can enhance the precision of RNA delivery [233].

Inorganic Nanomaterials: Inorganic nanomaterials, such as gold or silica nanoparticles, are emerging as alternative delivery systems for RNA therapeutics. These materials offer unique properties, including high stability and the ability to be engineered for specific applications [234]. For instance, gold nanoparticles can be functionalized with RNA molecules and used for targeted delivery or imaging applications [235]. Research into inorganic nanomaterials focuses on optimizing their biocompatibility, reducing potential toxicity, and enhancing their delivery efficiency. The development of inorganic nanomaterials for RNA delivery represents an exciting area of innovation with potential applications in diagnostics and therapeutics [236].

RNA-Based Delivery Systems: In addition to traditional delivery vehicles, next-generation RNA-based delivery systems are being explored. These systems include RNA carriers that can self-assemble into nanoparticles or other structures capable of efficiently delivering RNA therapeutics [237]. RNA carriers can be engineered to encapsulate RNA molecules, protect them from degradation, and facilitate their release within target cells [238]. Innovations in RNA-based delivery systems aim to address challenges related to stability, specificity, and cellular uptake. By leveraging the unique properties of RNA, these systems offer the potential for more effective and versatile RNA therapeutics [239].

#### 8.3.4. Integration with Digital Health Technologies

RNA Therapeutics and Digital Health: The integration of RNA therapeutics with digital health technologies represents a promising frontier for personalized medicine. Digital health technologies, such as wearable devices and remote monitoring systems, can provide real-time data on patient responses and therapeutic outcomes [240]. This information can be used to optimize RNA-based treatments and tailor them to individual patient needs. For example, digital health tools can monitor biomarkers or side effects in patients receiving RNA therapies, allowing for timely adjustments to treatment plans [241]. Additionally, the use of digital platforms for patient engagement and education can enhance adherence to RNA-based therapies and improve overall outcomes [242].

Data-Driven Drug Development: The integration of digital health technologies with RNA therapeutics can also facilitate data-driven drug development. By leveraging large datasets and advanced analytics, researchers can gain insights into the efficacy and safety of RNA-based therapies, identify new therapeutic targets, and accelerate the development of next-generation RNA therapeutics [243]. Efforts to integrate digital health technologies with RNA therapeutics are expected to drive innovation and improve the precision and effectiveness of RNA-based treatments [244]. The potential for next-generation RNA therapeutics is vast, with advancements in RNA technologies, novel therapeutic applications, and emerging delivery systems paving the way for new and transformative treatments. By addressing current challenges and embracing innovative approaches, the field of RNA therapeutics is poised for continued growth and impact. The integration of digital health technologies and data-driven approaches will further enhance the development and application of RNA-based therapies, offering the promise of personalized and effective treatments for a wide range of diseases.

## 9. Conclusions

### 9.1. Summary of Key Findings

The COVID-19 pandemic has significantly influenced the field of RNA therapeutics, accelerating technological advancements and highlighting both the potential and current limitations of these treatments. Here, we summarize the key findings from our review:

**1. Rapid Development and Success of mRNA Vaccines:** The pandemic underscored the transformative potential of mRNA vaccines. The swift development and global distribution of vaccines by Pfizer-BioNTech and Moderna showcased the ability of mRNA technology to respond rapidly to emergent health threats. The success of these vaccines demonstrated the efficacy of mRNA therapeutics and validated their role in combating infectious diseases.

**2. Surge in Lipid Nanoparticle (LNP) Technology:** Lipid nanoparticles emerged as a critical component in the delivery of mRNA vaccines. Their role in encapsulating RNA, protecting it from degradation, and facilitating its uptake into cells was pivotal. Innovations in LNP technology, such as improvements in lipid compositions and formulation strategies, were essential for achieving high efficacy and safety in mRNA vaccines.

**3. Exploration of Alternative Delivery Systems:** In addition to LNPs, various alternative delivery systems were developed to address specific challenges in RNA delivery. These included viral vectors, cell-penetrating peptides, and inorganic nanoparticles. Each system offers unique advantages, such as enhanced targeting or stability, and contributes to the expanding arsenal of tools for RNA therapeutics.

**4. Regulatory and Ethical Considerations:** The rapid pace of RNA therapeutic development during the pandemic highlighted the need for adaptive regulatory frameworks. Expedited approval processes were implemented to meet urgent public health needs, but this raised questions about balancing speed with comprehensive safety evaluations. Ethical issues, including vaccine equity and public trust, were also brought into focus, underscoring the importance of addressing these concerns in future therapeutic developments.

**5. Long-Term Implications and Future Directions:** The pandemic has laid a strong foundation for the future of RNA therapeutics. The advancements achieved in RNA technology and delivery systems provide a platform for further development. Future research will focus on overcoming existing challenges, such as improving delivery efficiency and reducing immunogenicity, while exploring new therapeutic applications.

### 9.2. The Future of RNA Therapeutics in a Post-Pandemic World

As the world moves beyond the pandemic, RNA therapeutics are poised to play a crucial role in addressing a broad range of medical conditions. The future of RNA therapeutics will be shaped by several key factors:

**1. Expansion Beyond Infectious Diseases:** The success of mRNA vaccines has demonstrated the potential of RNA technology for various therapeutic applications beyond infectious diseases. The focus is shifting towards using RNA therapeutics for cancer treatment, genetic disorders, and chronic diseases. Innovations in RNA-based therapies will aim to provide personalized treatment options by harnessing advances in genomics and precision medicine.

**2. Advancements in Delivery Technologies:** Improving RNA delivery systems remains a critical area of research. Future advancements will seek to enhance the efficiency and specificity of RNA delivery to target cells and tissues. This includes optimizing lipid nanoparticles, developing new polymer-based carriers, and exploring novel delivery mechanisms such as responsive or on-demand systems. The goal is to achieve more effective and safer RNA-based treatments.

**3. Integration with Digital Health Technologies:** The integration of RNA therapeutics with digital health technologies presents an opportunity for personalized medicine. Digital health tools, such as wearable devices and remote monitoring systems, can provide real-time data on patient responses and treatment outcomes. This information can be used to tailor RNA-based therapies to individual needs and improve overall treatment efficacy.

**4. Addressing Equity and Accessibility:** Ensuring equitable access to RNA therapeutics will be a major challenge in the post-pandemic world. Efforts must be made to address disparities in vaccine and therapy distribution, especially in low-resource settings. Building global infrastructure and addressing logistical challenges will be essential for making RNA-based treatments accessible to diverse populations.

**5. Continued Research and Innovation:** Ongoing research and innovation will be crucial for advancing the field of RNA therapeutics. This includes exploring new RNA technologies, optimizing delivery systems, and addressing safety and efficacy concerns. Collaboration between researchers, clinicians, and industry stakeholders will drive the development of next-generation RNA therapies and expand their applications.

### 9.3. Final Thoughts on the Role of RNA Technology in Global Health

RNA technology has emerged as a transformative force in global health, with the COVID-19 pandemic accelerating its adoption and development. The success of mRNA vaccines has validated the potential of RNA therapeutics to address urgent health challenges and has set the stage for future advancements. RNA technology offers the promise of more personalized and precise treatments, with applications extending beyond infectious diseases to include cancer, genetic disorders, and other complex conditions. As the field continues to evolve, it is essential to address remaining challenges, such as improving delivery systems and ensuring equitable access to treatments. The future of RNA therapeutics holds great promise for advancing global health. By leveraging ongoing research, embracing innovation, and addressing ethical and logistical challenges, RNA technology can contribute to more effective and accessible healthcare solutions. The lessons learned from the COVID-19 pandemic will guide the continued development and application of RNA-based therapies, ultimately leading to significant improvements in health outcomes worldwide.

## Figures and Tables

**Table 1:** Comparative analysis of advantages and disadvantages of different RNA delivery systems.

Delivery System	Advantages	Disadvantages
**Lipid Nanoparticles (LNPs)**	- High efficiency in RNA delivery due to effective endosomal escape [58] - Low immunogenicity, suitable for repeated administration [59] - Biocompatible and scalable [60]	- Potential allergic reactions, particularly to PEG component [61] - Accumulation in liver, which may lead to off-target effects
**Polymeric Nanoparticles**	- Made of biodegradable polymers like PLGA or PEI, offering good stability and biocompatibility [58] - Protect RNA from degradation	- Less efficient endosomal escape, limiting effective RNA delivery into the cytoplasm [58] - Often less effective compared to LNPs in cellular uptake and release
**Viral Vectors**	- High transduction efficiency, able to deliver RNA into a wide range of cell types [59] - Well-established in gene therapy applications	- Elicit strong immune responses, limiting repeated use [59] - Higher potential for causing immunogenicity, which can trigger adverse reactions or limit long-term therapy
**Exosomes**	- Natural nanoparticles with low immunogenicity and inherent ability to transfer RNA between cells - High biocompatibility [60]	- Limited scalability in production [60] - Difficult to manipulate for targeting specific tissues or cells - Less efficient compared to LNPs in large-scale therapeutic applications

## Data Availability

Not applicable.

## References

[1] Lee M.-J., Lee I., Wang K. (2022). Recent Advances in RNA Therapy and Its Carriers to Treat the Single-Gene Neurological Disorders. Biomedicines.

[2] Sayed N., Allawadhi P., Khurana A., Singh V., Navik U., Pasumarthi S.K., Khurana I., Banothu A.K., Weiskirchen R., Bharani K.K. (2022). Gene Therapy: Comprehensive Overview and Therapeutic Applications. Life Sci..

[3] Veiga N., Diesendruck Y., Peer D. (2020). Targeted Lipid Nanoparticles for RNA Therapeutics and Immunomodulation in Leukocytes. Adv. Drug Deliv. Rev..

[4] Verma A. (2018). Recent Advances in Antisense Oligonucleotide Therapy in Genetic Neuromuscular Diseases. Ann. Indian Acad. Neurol..

[5] Androsavich J.R. (2024). Frameworks for Transformational Breakthroughs in RNA-Based Medicines. Nat. Rev. Drug Discov..

[6] Chavda V., Soni S., Vora L., Soni S., Khadela A., Ajabiya J. (2022). MRNA-Based Vaccines and Therapeutics for COVID-19 and Future Pandemics. Vaccines.

[7] Bok K., Sitar S., Graham B.S., Mascola J.R. (2021). Accelerated COVID-19 Vaccine Development: Milestones, Lessons, and Prospects. Immunity.

[8] Liu T., Tian Y., Zheng A., Cui C. (2022). Design Strategies for and Stability of MRNA–Lipid Nanoparticle COVID-19 Vaccines. Polymers.

[9] Lokras A.G., Bobak T.R., Baghel S.S., Sebastiani F., Foged C. (2024). Advances in the Design and Delivery of RNA Vaccines for Infectious Diseases. Adv. Drug Deliv. Rev..

[10] Yetisgin A.A., Cetinel S., Zuvin M., Kosar A., Kutlu O. (2020). Therapeutic Nanoparticles and Their Targeted Delivery Applications. Molecules.

[11] Dhuri K., Bechtold C., Quijano E., Pham H., Gupta A., Vikram A., Bahal R. (2020). Antisense Oligonucleotides: An Emerging Area in Drug Discovery and Development. J. Clin. Med..

[12] Adams D., Gonzalez-Duarte A., O’Riordan W.D., Yang C.-C., Ueda M., Kristen A.V., Tournev I., Schmidt H.H., Coelho T., Berk J.L. (2018). Patisiran, an RNAi Therapeutic, for Hereditary Transthyretin Amyloidosis. N. Engl. J. Med..

[13] Zimmermann T.S., Karsten V., Chan A., Chiesa J., Boyce M., Bettencourt B.R., Hutabarat R., Nochur S., Vaishnaw A., Gollob J. (2017). Clinical Proof of Concept for a Novel Hepatocyte-Targeting GalNAc-SiRNA Conjugate. Mol. Ther..

[14] Garber K. (2016). Alnylam Terminates Revusiran Program, Stock Plunges. Nat. Biotechnol..

[15] Zorde Khvalevsky E., Gabai R., Rachmut I.H., Horwitz E., Brunschwig Z., Orbach A., Shemi A., Golan T., Domb A.J., Yavin E. (2013). Mutant KRAS Is a Druggable Target for Pancreatic Cancer. Proc. Natl. Acad. Sci. USA.

[16] Titze-de-Almeida R., David C., Titze-de-Almeida S.S. (2017). The Race of 10 Synthetic RNAi-Based Drugs to the Pharmaceutical Market. Pharm. Res..

[17] Zhu Y., Zhu L., Wang X., Jin H. (2022). RNA-Based Therapeutics: An Overview and Prospectus. Cell Death Dis..

[18] Anthony K. (2022). RNA-Based Therapeutics for Neurological Diseases. RNA Biol..

[19] Le Huy B., Bui Thi Phuong H., Luong Xuan H. (2024). Advantages and Disadvantages of RNA Therapeutics. Prog. Mol. Biol. Transl. Sci..

[20] Scioli Montoto S., Muraca G., Ruiz M.E. (2020). Solid Lipid Nanoparticles for Drug Delivery: Pharmacological and Biopharmaceutical Aspects. Front. Mol. Biosci..

[21] Ghassemi K.M., Demelenne A., Crommen J., Servais A.-C., Fillet M. (2022). Improvement of Chemo- and Stereoselectivity for Phosphorothioate Oligonucleotides in Capillary Electrophoresis by Addition of Cyclodextrins. J. Chromatogr. A.

[22] Yang L., Gong L., Wang P., Zhao X., Zhao F., Zhang Z., Li Y., Huang W. (2022). Recent Advances in Lipid Nanoparticles for Delivery of MRNA. Pharmaceutics.

[23] Kim S., Choi B., Kim Y., Shim G. (2023). Immune-Modulating Lipid Nanomaterials for the Delivery of Biopharmaceuticals. Pharmaceutics.

[24] Chavda V.P., Jogi G., Dave S., Patel B.M., Vineela Nalla L., Koradia K. (2023). MRNA-Based Vaccine for COVID-19: They Are New but Not Unknown!. Vaccines.

[25] Zhang J., Liu Y., Li C., Xiao Q., Zhang D., Chen Y., Rosenecker J., Ding X., Guan S. (2023). Recent Advances and Innovations in the Preparation and Purification of In Vitro-Transcribed-MRNA-Based Molecules. Pharmaceutics.

[26] Alameh M.-G., Tombácz I., Bettini E., Lederer K., Ndeupen S., Sittplangkoon C., Wilmore J.R., Gaudette B.T., Soliman O.Y., Pine M. (2021). Lipid Nanoparticles Enhance the Efficacy of MRNA and Protein Subunit Vaccines by Inducing Robust T Follicular Helper Cell and Humoral Responses. Immunity.

[27] Li Y., Wang M., Peng X., Yang Y., Chen Q., Liu J., She Q., Tan J., Lou C., Liao Z. (2023). MRNA Vaccine in Cancer Therapy: Current Advance and Future Outlook. Clin. Transl. Med..

[28] Zahedipour F., Zahedipour F., Zamani P., Jaafari M.R., Sahebkar A. (2024). Harnessing CRISPR Technology for Viral Therapeutics and Vaccines: From Preclinical Studies to Clinical Applications. Virus Res..

[29] Mo C., Li X., Wu Q., Fan Y., Liu D., Zhu Y., Yang Y., Liao X., Zhou Z., Zhou L. (2023). SARS-CoV-2 MRNA Vaccine Requires Signal Peptide to Induce Antibody Responses. Vaccine.

[30] Szabó G.T., Mahiny A.J., Vlatkovic I. (2022). COVID-19 MRNA Vaccines: Platforms and Current Developments. Mol. Ther..

[31] Qin S., Tang X., Chen Y., Chen K., Fan N., Xiao W., Zheng Q., Li G., Teng Y., Wu M. (2022). MRNA-Based Therapeutics: Powerful and Versatile Tools to Combat Diseases. Signal Transduct. Target. Ther..

[32] Wilson B., Geetha K.M. (2022). Lipid Nanoparticles in the Development of MRNA Vaccines for COVID-19. J. Drug Deliv. Sci. Technol..

[33] Tang X., Zhang Y., Han X. (2023). Ionizable Lipid Nanoparticles for MRNA Delivery. Adv. NanoBiomed Res..

[34] Zhang T., Yin H., Li Y., Yang H., Ge K., Zhang J., Yuan Q., Dai X., Naeem A., Weng Y. (2024). Optimized Lipid Nanoparticles (LNPs) for Organ-Selective Nucleic Acids Delivery in Vivo. iScience.

[35] Han J., Lim J., Wang C.-P.J., Han J.-H., Shin H.E., Kim S.-N., Jeong D., Lee S.H., Chun B.-H., Park C.G. (2023). Lipid Nanoparticle-Based MRNA Delivery Systems for Cancer Immunotherapy. Nano Converg..

[36] Lee Y., Jeong M., Park J., Jung H., Lee H. (2023). Immunogenicity of Lipid Nanoparticles and Its Impact on the Efficacy of MRNA Vaccines and Therapeutics. Exp. Mol. Med..

[37] Knezevic I., Liu M.A., Peden K., Zhou T., Kang H.-N. (2021). Development of MRNA Vaccines: Scientific and Regulatory Issues. Vaccines.

[38] Puranik A., Lenehan P.J., Silvert E., Niesen M.J.M., Corchado-Garcia J., O’Horo J.C., Virk A., Swift M.D., Gordon J.E., Speicher L.L. (2022). Comparative Effectiveness of MRNA-1273 and BNT162b2 against Symptomatic SARS-CoV-2 Infection. Med.

[39] Barbier A.J., Jiang A.Y., Zhang P., Wooster R., Anderson D.G. (2022). The Clinical Progress of MRNA Vaccines and Immunotherapies. Nat. Biotechnol..

[40] Fang E., Liu X., Li M., Zhang Z., Song L., Zhu B., Wu X., Liu J., Zhao D., Li Y. (2022). Advances in COVID-19 MRNA Vaccine Development. Signal Transduct. Target. Ther..

[41] Watson O.J., Barnsley G., Toor J., Hogan A.B., Winskill P., Ghani A.C. (2022). Global Impact of the First Year of COVID-19 Vaccination: A Mathematical Modelling Study. Lancet Infect. Dis..

[42] Oude Blenke E., Örnskov E., Schöneich C., Nilsson G.A., Volkin D.B., Mastrobattista E., Almarsson Ö., Crommelin D.J.A. (2023). The Storage and In-Use Stability of MRNA Vaccines and Therapeutics: Not A Cold Case. J. Pharm. Sci..

[43] Uddin M.N., Roni M.A. (2021). Challenges of Storage and Stability of MRNA-Based COVID-19 Vaccines. Vaccines.

[44] da Fonseca E.M., Shadlen K.C., Achcar H. (2023). de M. Vaccine Technology Transfer in a Global Health Crisis: Actors, Capabilities, and Institutions. Res. Policy.

[45] Goyal F., Chattopadhyay A., Navik U., Jain A., Reddy P.H., Bhatti G.K., Bhatti J.S. (2024). Advancing Cancer Immunotherapy: The Potential of MRNA Vaccines As a Promising Therapeutic Approach. Adv. Ther..

[46] Jeong M., Lee Y., Park J., Jung H., Lee H. (2023). Lipid Nanoparticles (LNPs) for in Vivo RNA Delivery and Their Breakthrough Technology for Future Applications. Adv. Drug Deliv. Rev..

[47] Han X., Gong N., Xue L., Billingsley M.M., El-Mayta R., Shepherd S.J., Alameh M.-G., Weissman D., Mitchell M.J. (2023). Ligand-Tethered Lipid Nanoparticles for Targeted RNA Delivery to Treat Liver Fibrosis. Nat. Commun..

[48] Wu L., Li X., Qian X., Wang S., Liu J., Yan J. (2024). Lipid Nanoparticle (LNP) Delivery Carrier-Assisted Targeted Controlled Release MRNA Vaccines in Tumor Immunity. Vaccines.

[49] Friis K.P., Gracin S., Oag S., Leijon A., Sand E., Lindberg B., Lázaro-Ibáñez E., Lindqvist J., Whitehead K.A., Bak A. (2023). Spray Dried Lipid Nanoparticle Formulations Enable Intratracheal Delivery of MRNA. J. Control Release.

[50] Mukai H., Ogawa K., Kato N., Kawakami S. (2022). Recent Advances in Lipid Nanoparticles for Delivery of Nucleic Acid, MRNA, and Gene Editing-Based Therapeutics. Drug Metab. Pharmacokinet..

[51] Berger M., Degey M., Leblond Chain J., Maquoi E., Evrard B., Lechanteur A., Piel G. (2023). Effect of PEG Anchor and Serum on Lipid Nanoparticles: Development of a Nanoparticles Tracking Method. Pharmaceutics.

[52] Kutikuppala L.V.S., Kourampi I., Kanagala R.S.D., Bhattacharjee P., Boppana S.H. (2024). Prospects and Challenges in Developing MRNA Vaccines for Infectious Diseases and Oncogenic Viruses. Med. Sci..

[53] Han X., Zhang H., Butowska K., Swingle K.L., Alameh M.-G., Weissman D., Mitchell M.J. (2021). An Ionizable Lipid Toolbox for RNA Delivery. Nat. Commun..

[54] Hashiba K., Sato Y., Harashima H. (2017). PH-Labile PEGylation of SiRNA-Loaded Lipid Nanoparticle Improves Active Targeting and Gene Silencing Activity in Hepatocytes. J. Control Release.

[55] Zhang J., Ali K., Wang J. (2024). Research Advances of Lipid Nanoparticles in the Treatment of Colorectal Cancer. Int. J. Nanomed..

[56] Chan C., Du S., Dong Y., Cheng X. (2021). Computational and Experimental Approaches to Investigate Lipid Nanoparticles as Drug and Gene Delivery Systems. Curr. Top. Med. Chem..

[57] Lu Y., Huang W., Li M., Zheng A. (2023). Exosome-Based Carrier for RNA Delivery: Progress and Challenges. Pharmaceutics.

[58] Blakney A.K., McKay P.F., Hu K., Samnuan K., Jain N., Brown A., Thomas A., Rogers P., Polra K., Sallah H. (2021). Polymeric and Lipid Nanoparticles for Delivery of Self-Amplifying RNA Vaccines. J. Control Release.

[59] Li X., Le Y., Zhang Z., Nian X., Liu B., Yang X. (2023). Viral Vector-Based Gene Therapy. Int. J. Mol. Sci..

[60] Zhang W., Jiang Y., He Y., Boucetta H., Wu J., Chen Z., He W. (2023). Lipid Carriers for MRNA Delivery. Acta Pharm. Sin. B.

[61] Guerrini G., Gioria S., Sauer A.V., Lucchesi S., Montagnani F., Pastore G., Ciabattini A., Medaglini D., Calzolai L. (2022). Monitoring Anti-PEG Antibodies Level upon Repeated Lipid Nanoparticle-Based COVID-19 Vaccine Administration. Int. J. Mol. Sci..

[62] Kremsner P.G., Mann P., Kroidl A., Leroux-Roels I., Schindler C., Gabor J.J., Schunk M., Leroux-Roels G., Bosch J.J., Fendel R. (2021). Safety and Immunogenicity of an MRNA-Lipid Nanoparticle Vaccine Candidate against SARS-CoV-2. Wien. Klin. Wochenschr..

[63] Moghimi S.M. (2021). Allergic Reactions and Anaphylaxis to LNP-Based COVID-19 Vaccines. Mol. Ther..

[64] Berger M., Toussaint F., Djemaa S.B., Laloy J., Pendeville H., Evrard B., Jerôme C., Lechanteur A., Mottet D., Debuigne A. (2023). Poly(Vinyl Pyrrolidone) Derivatives as PEG Alternatives for Stealth, Non-Toxic and Less Immunogenic SiRNA-Containing Lipoplex Delivery. J. Control Release.

[65] Verbeke R., Hogan M.J., Loré K., Pardi N. (2022). Innate Immune Mechanisms of MRNA Vaccines. Immunity.

[66] Gote V., Bolla P.K., Kommineni N., Butreddy A., Nukala P.K., Palakurthi S.S., Khan W. (2023). A Comprehensive Review of MRNA Vaccines. Int. J. Mol. Sci..

[67] Chehelgerdi M., Chehelgerdi M. (2023). The Use of RNA-Based Treatments in the Field of Cancer Immunotherapy. Mol. Cancer.

[68] Arjunan P., Kathirvelu D., Mahalingam G., Goel A.K., Zacharaiah U.G., Srivastava A., Marepally S. (2024). Lipid-Nanoparticle-Enabled Nucleic Acid Therapeutics for Liver Disorders. Acta Pharm. Sin. B.

[69] Jiang X., Abedi K., Shi J. (2023). Polymeric Nanoparticles for RNA Delivery. Encyclopedia of Nanomaterials.

[70] Alsaab H.O., Alharbi F.D., Alhibs A.S., Alanazi N.B., Alshehri B.Y., Saleh M.A., Alshehri F.S., Algarni M.A., Almugaiteeb T., Uddin M.N. (2022). PLGA-Based Nanomedicine: History of Advancement and Development in Clinical Applications of Multiple Diseases. Pharmaceutics.

[71] Kim S., No Y.H., Sluyter R., Konstantinov K., Kim Y.H., Kim J.H. (2024). Peptide-Nanoparticle Conjugates as a Theranostic Platform. Coord. Chem. Rev..

[72] Qiu C., Xia F., Zhang J., Shi Q., Meng Y., Wang C., Pang H., Gu L., Xu C., Guo Q. (2023). Advanced Strategies for Overcoming Endosomal/Lysosomal Barrier in Nanodrug Delivery. Research.

[73] Digiacomo L., Renzi S., Pirrottina A., Amenitsch H., De Lorenzi V., Pozzi D., Cardarelli F., Caracciolo G. (2024). PEGylation-Dependent Cell Uptake of Lipid Nanoparticles Revealed by Spatiotemporal Correlation Spectroscopy. ACS Pharmacol. Transl. Sci..

[74] Travieso T., Li J., Mahesh S., Mello J.D.F.R.E., Blasi M. (2022). The Use of Viral Vectors in Vaccine Development. Npj Vaccines.

[75] Chang J. (2021). Adenovirus Vectors: Excellent Tools for Vaccine Development. Immune Netw..

[76] Verdera H.C., Kuranda K., Mingozzi F. (2020). AAV Vector Immunogenicity in Humans: A Long Journey to Successful Gene Transfer. Mol. Ther..

[77] Chavda V., Bezbaruah R., Valu D., Patel B., Kumar A., Prasad S., Kakoti B., Kaushik A., Jesawadawala M. (2023). Adenoviral Vector-Based Vaccine Platform for COVID-19: Current Status. Vaccines.

[78] Dobrowsky T., Gianni D., Pieracci J., Suh J. (2021). AAV Manufacturing for Clinical Use: Insights on Current Challenges from the Upstream Process Perspective. Curr. Opin. Biomed. Eng..

[79] Bulcha J.T., Wang Y., Ma H., Tai P.W.L., Gao G. (2021). Viral Vector Platforms within the Gene Therapy Landscape. Signal Transduct. Target. Ther..

[80] Kiaie S.H., Majidi Zolbanin N., Ahmadi A., Bagherifar R., Valizadeh H., Kashanchi F., Jafari R. (2022). Recent Advances in MRNA-LNP Therapeutics: Immunological and Pharmacological Aspects. J. Nanobiotechnol..

[81] Yang W., Mixich L., Boonstra E., Cabral H. (2023). Polymer-Based MRNA Delivery Strategies for Advanced Therapies. Adv. Healthc. Mater..

[82] Marques A.C., Costa P.C., Velho S., Amaral M.H. (2023). Lipid Nanoparticles Functionalized with Antibodies for Anticancer Drug Therapy. Pharmaceutics.

[83] Clemente B., Denis M., Silveira C.P., Schiavetti F., Brazzoli M., Stranges D. (2023). Straight to the Point: Targeted MRNA-Delivery to Immune Cells for Improved Vaccine Design. Front. Immunol..

[84] Paunovska K., Loughrey D., Dahlman J.E. (2022). Drug Delivery Systems for RNA Therapeutics. Nat. Rev. Genet..

[85] Chen P., Cabral H. (2024). Enhancing Targeted Drug Delivery through Cell-Specific Endosomal Escape. Chem. Med. Chem..

[86] Thi H.V., Thi L.-A.N., Tang T.L., Chu D.-T. (2024). Biosafety and Regulatory Issues of RNA Therapeutics. Prog. Mol Biol. Transl. Sci..

[87] Wang B., Pei J., Xu S., Liu J., Yu J. (2023). Recent Advances in MRNA Cancer Vaccines: Meeting Challenges and Embracing Opportunities. Front. Immunol..

[88] Ni L. (2023). Advances in MRNA-Based Cancer Vaccines. Vaccines.

[89] Weber J.S., Carlino M.S., Khattak A., Meniawy T., Ansstas G., Taylor M.H., Kim K.B., McKean M., Long G.V., Sullivan R.J. (2024). Individualised Neoantigen Therapy MRNA-4157 (V940) plus Pembrolizumab versus Pembrolizumab Monotherapy in Resected Melanoma (KEYNOTE-942): A Randomised, Phase 2b Study. Lancet.

[90] Cowzer D., Zameer M., Conroy M., Kolch W., Duffy A.G. (2022). Targeting KRAS in Pancreatic Cancer. J. Pers. Med..

[91] Zhang X., Hai L., Gao Y., Yu G., Sun Y. (2023). Lipid Nanomaterials-Based RNA Therapy and Cancer Treatment. Acta Pharm. Sin. B.

[92] Li Q. (2020). Nusinersen as a Therapeutic Agent for Spinal Muscular Atrophy. Yonsei Med. J..

[93] Yuan M., Han Z., Liang Y., Sun Y., He B., Chen W., Li F. (2023). MRNA Nanodelivery Systems: Targeting Strategies and Administration Routes. Biomater. Res..

[94] Yonezawa S., Koide H., Asai T. (2020). Recent Advances in SiRNA Delivery Mediated by Lipid-Based Nanoparticles. Adv. Drug Deliv. Rev..

[95] Wilkinson M.J., Bajaj A., Brousseau M.E., Taub P.R. (2024). Harnessing RNA Interference for Cholesterol Lowering: The Bench-to-Bedside Story of Inclisiran. J. Am. Heart Assoc..

[96] Kishore R., Magadum A. (2024). Cell-Specific MRNA Therapeutics for Cardiovascular Diseases and Regeneration. J. Cardiovasc. Dev. Dis..

[97] Kackos C.M., DeBeauchamp J., Davitt C.J.H., Lonzaric J., Sealy R.E., Hurwitz J.L., Samsa M.M., Webby R.J. (2023). Seasonal Quadrivalent MRNA Vaccine Prevents and Mitigates Influenza Infection. Npj Vaccines.

[98] Borkens Y. (2023). Malaria & MRNA Vaccines: A Possible Salvation from One of the Most Relevant Infectious Diseases of the Global South. Acta Parasitol..

[99] Gareri C., Polimeni A., Giordano S., Tammè L., Curcio A., Indolfi C. (2022). Antisense Oligonucleotides and Small Interfering RNA for the Treatment of Dyslipidemias. J. Clin. Med..

[100] Kiernan M.G., Coffey J.C., Sahebally S.M., Tibbitts P., Lyons E.M., O’leary E., Owolabi F., Dunne C.P. (2020). Systemic Molecular Mediators of Inflammation Differentiate Between Crohn’s Disease and Ulcerative Colitis, Implicating Threshold Levels of IL-10 and Relative Ratios of Pro-Inflammatory Cytokines in Therapy. J. Crohn’s Colitis.

[101] Xie N., Shen G., Gao W., Huang Z., Huang C., Fu L. (2023). Neoantigens: Promising Targets for Cancer Therapy. Signal Transduct. Target. Ther..

[102] Tian Z., Liang G., Cui K., Liang Y., Wang Q., Lv S., Cheng X., Zhang L. (2021). Insight Into the Prospects for RNAi Therapy of Cancer. Front. Pharmacol..

[103] May M. (2024). How MRNA Is Powering a Personalized Vaccine Revolution. Nat. Med..

[104] Lorentzen C.L., Haanen J.B., Met Ö., Svane I.M. (2022). Clinical Advances and Ongoing Trials of MRNA Vaccines for Cancer Treatment. Lancet Oncol..

[105] Edinoff A.N., Nguyen L.H., Odisho A.S., Maxey B.S., Pruitt J.W., Girma B., Cornett E.M., Kaye A.M., Kaye A.D. (2021). The Antisense Oligonucleotide Nusinersen for Treatment of Spinal Muscular Atrophy. Orthop. Rev..

[106] Rowe S.M., Zuckerman J.B., Dorgan D., Lascano J., McCoy K., Jain M., Schechter M.S., Lommatzsch S., Indihar V., Lechtzin N. (2023). Inhaled MRNA Therapy for Treatment of Cystic Fibrosis: Interim Results of a Randomized, Double-blind, Placebo-controlled Phase 1/2 Clinical Study. J. Cyst. Fibros..

[107] Ansori A.N., Antonius Y., Susilo R.J., Hayaza S., Kharisma V.D., Parikesit A.A., Zainul R.J., Jakhmola V., Saklani T., Rebezov M. (2023). Application of CRISPR-Cas9 Genome Editing Technology in Various Fields: A Review. Narra. J..

[108] Perez-Garcia C.G., Diaz-Trelles R., Vega J.B., Bao Y., Sablad M., Limphong P., Chikamatsu S., Yu H., Taylor W., Karmali P.P. (2022). Development of an MRNA Replacement Therapy for Phenylketonuria. Mol. Ther. Nucleic Acids.

[109] Sinning D., Landmesser U. (2020). Low-Density Lipoprotein-Cholesterol Lowering Strategies for Prevention of Atherosclerotic Cardiovascular Disease: Focus on SiRNA Treatment Targeting PCSK9 (Inclisiran). Curr. Cardiol. Rep..

[110] Traber G.M., Yu A.-M. (2023). RNAi-Based Therapeutics and Novel RNA Bioengineering Technologies. J. Pharmacol. Exp. Ther..

[111] Cooke J.P., Youker K.A. (2022). Future Impact of MRNA Therapy on Cardiovascular Diseases. Methodist Debakey Cardiovasc. J..

[112] Abdul-Rahman T., Lizano-Jubert I., Bliss Z.S.B., Garg N., Meale E., Roy P., Crino S.A., Deepak B.L., Miteu G.D., Wireko A.A. (2024). RNA in Cardiovascular Disease: A New Frontier of Personalized Medicine. Prog. Cardiovasc. Dis..

[113] Jung H.N., Lee S.-Y., Lee S., Youn H., Im H.-J. (2022). Lipid Nanoparticles for Delivery of RNA Therapeutics: Current Status and the Role of in Vivo Imaging. Theranostics.

[114] Ewaisha R., Anderson K.S. (2023). Immunogenicity of CRISPR Therapeutics—Critical Considerations for Clinical Translation. Front. Bioeng. Biotechnol..

[115] Hostiuc M., Scafa A., Iancu B., Iancu D., Isailă O.-M., Ion O.M., Stroe A., Diaconu C., Epistatu D., Hostiuc S. (2024). Ethical Implications of Developing RNA-Based Therapies for Cardiovascular Disorders. Front. Bioeng. Biotechnol..

[116] Mohammed I., Nauman A., Paul P., Ganesan S., Chen K.-H., Jalil S.M.S., Jaouni S.H., Kawas H., Khan W.A., Vattoth A.L. (2022). The Efficacy and Effectiveness of the COVID-19 Vaccines in Reducing Infection, Severity, Hospitalization, and Mortality: A Systematic Review. Hum. Vaccin. Immunother..

[117] Reynolds L., Dewey C., Asfour G., Little M. (2023). Vaccine Efficacy against SARS-CoV-2 for Pfizer BioNTech, Moderna, and AstraZeneca Vaccines: A Systematic Review. Front. Public Health.

[118] Nitika, Wei J., Hui A.-M. (2021). The Development of MRNA Vaccines for Infectious Diseases: Recent Updates. Infect. Drug Resist..

[119] Tian Y., Deng Z., Yang P. (2022). MRNA Vaccines: A Novel Weapon to Control Infectious Diseases. Front. Microbiol..

[120] Shartouny J.R., Lowen A.C. (2022). Message in a Bottle: MRNA Vaccination for Influenza. J. Gen. Virol..

[121] Md Khairi L.N.H., Fahrni M.L., Lazzarino A.I. (2022). The Race for Global Equitable Access to COVID-19 Vaccines. Vaccines.

[122] Liu C., Papukashvili D., Dong Y., Wang X., Hu X., Yang N., Cai J., Xie F., Rcheulishvili N., Wang P.G. (2022). Identification of Tumor Antigens and Design of MRNA Vaccine for Colorectal Cancer Based on the Immune Subtype. Front. Cell Dev. Biol..

[123] Seclì L., Leoni G., Ruzza V., Siani L., Cotugno G., Scarselli E., D’Alise A.M. (2023). Personalized Cancer Vaccines Go Viral: Viral Vectors in the Era of Personalized Immunotherapy of Cancer. Int. J. Mol. Sci..

[124] Movahed F., Darzi S., Mahdavi P., Salih Mahdi M., Qutaiba B., Allela O., Naji Sameer H., Adil M., Zarkhah H., Yasamineh S. (2024). The Potential Use of Therapeutics and Prophylactic MRNA Vaccines in Human Papillomavirus (HPV). Virol. J..

[125] Kong B., Kim Y., Kim E.H., Suk J.S., Yang Y. (2023). MRNA: A Promising Platform for Cancer Immunotherapy. Adv. Drug Deliv. Rev..

[126] Soroudi S., Jaafari M.R., Arabi L. (2024). Lipid Nanoparticle (LNP) Mediated MRNA Delivery in Cardiovascular Diseases: Advances in Genome Editing and CAR T Cell Therapy. J. Control Release.

[127] Lee S., Lee J., Cho S.-H., Roh G., Park H.-J., Lee Y.-J., Jeon H.-E., Lee Y.-S., Bae S.-H., Youn S.B. (2024). Assessing the Impact of MRNA Vaccination in Chronic Inflammatory Murine Model. Npj Vaccines.

[128] Aljabali A.A.A., Bashatwah R.M., Obeid M.A., Mishra V., Mishra Y., Serrano-Aroca Á., Lundstrom K., Tambuwala M.M. (2023). Current State of, Prospects for, and Obstacles to MRNA Vaccine Development. Drug Discov. Today.

[129] Towett G., Snead R.S., Grigoryan K., Marczika J. (2023). Geographical and Practical Challenges in the Implementation of Digital Health Passports for Cross-Border COVID-19 Pandemic Management: A Narrative Review and Framework for Solutions. Glob. Health.

[130] Ye Y., Zhang Q., Wei X., Cao Z., Yuan H.-Y., Zeng D.D. (2022). Equitable Access to COVID-19 Vaccines Makes a Life-Saving Difference to All Countries. Nat. Hum. Behav..

[131] Tran A., Witek T.J. (2021). The Emergency Use Authorization of Pharmaceuticals: History and Utility During the COVID-19 Pandemic. Pharmaceut. Med..

[132] Rizk J.G., Forthal D.N., Kalantar-Zadeh K., Mehra M.R., Lavie C.J., Rizk Y., Pfeiffer J.P., Lewin J.C. (2021). Expanded Access Programs, Compassionate Drug Use, and Emergency Use Authorizations during the COVID-19 Pandemic. Drug Discov. Today.

[133] Wong J.C., Lao C.T., Yousif M.M., Luga J.M. (2022). Fast Tracking—Vaccine Safety, Efficacy, and Lessons Learned: A Narrative Review. Vaccines.

[134] Stewart J., Honig P., AlJuburi L., Autor D., Berger S., Brady P., Fitton H., Garner C., Garvin M., Hukkelhoven M. (2021). COVID-19: A Catalyst to Accelerate Global Regulatory Transformation. Clin. Pharmacol. Ther..

[135] Skerritt J.H., Tucek-Szabo C., Sutton B., Nolan T. (2024). The Platform Technology Approach to MRNA Product Development and Regulation. Vaccines.

[136] Lu Y., Lindaas A., Matuska K., Izurieta H.S., McEvoy R., Menis M., Shi X., Steele W.R., Wernecke M., Chillarige Y. (2024). Real-World Effectiveness of MRNA COVID-19 Vaccines Among US Nursing Home Residents Aged ≥65 Years in the Pre-Delta and High Delta Periods. Open Forum Infect. Dis..

[137] Bruxvoort K.J., Sy L.S., Qian L., Ackerson B.K., Luo Y., Lee G.S., Tian Y., Florea A., Takhar H.S., Tubert J.E. (2022). Real-World Effectiveness of the MRNA-1273 Vaccine against COVID-19: Interim Results from a Prospective Observational Cohort Study. Lancet Reg. Health—Am..

[138] Verkerk K., Voest E.E. (2024). Generating and Using Real-World Data: A Worthwhile Uphill Battle. Cell.

[139] Zhou L.-Y., Qin Z., Zhu Y.-H., He Z.-Y., Xu T. (2019). Current RNA-Based Therapeutics in Clinical Trials. Curr. Gene Ther..

[140] Winch G.M., Cao D., Maytorena-Sanchez E., Pinto J., Sergeeva N., Zhang S. (2021). Operation Warp Speed: Projects Responding to the COVID-19 Pandemic. Proj. Leadersh. Soc..

[141] Sandbrink J.B., Koblentz G.D. (2022). Biosecurity Risks Associated with Vaccine Platform Technologies. Vaccine.

[142] Chirico F., Teixeira da Silva J.A. (2023). Evidence-Based Policies in Public Health to Address COVID-19 Vaccine Hesitancy. Future Virol..

[143] Asaduzzaman M., Khai T.S., de Claro V., Zaman F. (2023). Global Disparities in COVID-19 Vaccine Distribution: A Call for More Integrated Approaches to Address Inequities in Emerging Health Challenges. Challenges.

[144] Excler J.-L., Saville M., Privor-Dumm L., Gilbert S., Hotez P.J., Thompson D., Abdool-Karim S., Kim J.H. (2023). Factors, Enablers and Challenges for COVID-19 Vaccine Development. BMJ Glob. Health.

[145] Black S.B., Chandler R.E., Edwards K.M., Sturkenboom M.C.J.M. (2023). Assessing Vaccine Safety during a Pandemic: Recent Experience and Lessons Learned for the Future. Vaccine.

[146] Saw P.E., Song E. (2024). Advancements in Clinical RNA Therapeutics: Present Developments and Prospective Outlooks. Cell Rep. Med..

[147] Sparmann A., Vogel J. (2023). RNA-based Medicine: From Molecular Mechanisms to Therapy. EMBO J..

[148] Soumyanarayanan U., Choong M., Leong J., Lumpkin M.M., Rasi G., Skerritt J.H., Vogel S., Lim J.C.W. (2021). The COVID-19 Crisis as an Opportunity to Strengthen Global Regulatory Coordination for Sustained Enhanced Access to Diagnostics and Therapeutics. Clin. Transl. Sci..

[149] Jalilian H., Amraei M., Javanshir E., Jamebozorgi K., Faraji-Khiavi F. (2023). Ethical Considerations of the Vaccine Development Process and Vaccination: A Scoping Review. BMC Health Serv. Res..

[150] Chongwe G., Ali J., Kaye D.K., Michelo C., Kass N.E. (2023). Ethics of Adaptive Designs for Randomized Controlled Trials. Ethics Hum. Res..

[151] Cole A., Webster P., Van Liew D., Salas M., Aimer O., Malikova M.A. (2022). Safety Surveillance and Challenges in Accelerated COVID-19 Vaccine Development. Ther. Adv. Drug Saf..

[152] Calder T., Tong T., Hu D.J., Kim J.H., Kotloff K.L., Koup R.A., Marovich M.A., McElrath M.J., Read S.W., Robb M.L. (2022). Leveraging Lessons Learned from the COVID-19 Pandemic for HIV. Commun. Med..

[153] von Achenbach J. (2023). The Global Distribution of COVID-19 Vaccines by the Public-Private Partnership COVAX from a Public-Law Perspective. Leiden J. Int. Law.

[154] Das J.K., Chee H.Y., Lakhani S., Khan M.H., Islam M., Muhammad S., Bhutta Z.A. (2022). COVID-19 Vaccines: How Efficient and Equitable Was the Initial Vaccination Process?. Vaccines.

[155] Chapman L.A.C., Shukla P., Rodríguez-Barraquer I., Shete P.B., León T.M., Bibbins-Domingo K., Rutherford G.W., Schechter R., Lo N.C. (2022). Risk Factor Targeting for Vaccine Prioritization during the COVID-19 Pandemic. Sci. Rep..

[156] Jecker N.S., Wightman A.G., Diekema D.S. (2021). Vaccine Ethics: An Ethical Framework for Global Distribution of COVID-19 Vaccines. J. Med. Ethics.

[157] Corey L., Miner M.D. (2022). Accelerating Clinical Trial Development in Vaccinology: COVID-19 and Beyond. Curr. Opin. Immunol..

[158] Krause P.R., Fleming T.R., Ellenberg S.S., Henao-Restrepo A.M., Krause P., Fleming T., Alejandria M., Bhargava B., Ellenberg S., Garcia P. (2020). Maintaining Confidentiality of Emerging Results in COVID-19 Vaccine Trials Is Essential. Lancet.

[159] Jamrozik E., Selgelid M.J. (2020). COVID-19 Human Challenge Studies: Ethical Issues. Lancet Infect. Dis..

[160] Kumar V., Aranha V., Rajgarhia R., Royal A., Mehta K. (2021). Expanded Principles of Ethics and Its Implementation during COVID-19 Vaccine Trials: A Scoping Evidence Based Research Synthesis. Hum. Vaccin. Immunother..

[161] López-Camacho C., Alameh M.G., Peer D., Culis P. (2024). Editorial: RNA Vaccines for Prevalent and Newly Emerging Diseases. Front. Immunol..

[162] Kashte S., Gulbake A., El-Amin III S.F., Gupta A. (2021). COVID-19 Vaccines: Rapid Development, Implications, Challenges and Future Prospects. Hum. Cell.

[163] García L.Y., Cerda A.A. (2020). Contingent Assessment of the COVID-19 Vaccine. Vaccine.

[164] Kairuz D., Samudh N., Ely A., Arbuthnot P., Bloom K. (2022). Advancing MRNA Technologies for Therapies and Vaccines: An African Context. Front. Immunol..

[165] Youssef M., Hitti C., Puppin Chaves Fulber J., Kamen A.A. (2023). Enabling MRNA Therapeutics: Current Landscape and Challenges in Manufacturing. Biomolecules.

[166] Daniel S., Kis Z., Kontoravdi C., Shah N. (2022). Quality by Design for Enabling RNA Platform Production Processes. Trends Biotechnol..

[167] Hodel K.V.S., Fiuza B.S.D., Conceição R.S., Aleluia A.C.M., Pitanga T.N., Fonseca L.M.d.S., Valente C.O., Minafra-Rezende C.S., Machado B.A.S. (2024). Pharmacovigilance in Vaccines: Importance, Main Aspects, Perspectives, and Challenges—A Narrative Review. Pharmaceuticals.

[168] Burns L., Le Roux N., Kalesnik-Orszulak R., Christian J., Dudinak J., Rockhold F., Khozin S., O’Donnell J. (2023). Real-World Evidence for Regulatory Decision-Making: Updated Guidance from around the World. Front. Med..

[169] Shau W.-Y., Setia S., Shinde S., Santoso H., Furtner D. (2023). Generating Fit-for-Purpose Real-World Evidence in Asia: How Far Are We from Closing the Gaps?. Perspect. Clin. Res..

[170] Hamza N., Kulkarni U. (2022). A Narrative Review of the Challenges, Ethical Frameworks, and Guidelines in the Setting of COVID-19 Healthcare and Research. Perspect. Clin. Res..

[171] Lee H.S. (2022). Ethical Issues in Clinical Research and Publication. Kosin Med. J..

[172] Zimmerman T., Shiroma K., Fleischmann K.R., Xie B., Jia C., Verma N., Lee M.K. (2023). Misinformation and COVID-19 Vaccine Hesitancy. Vaccine.

[173] Hastings M.L., Krainer A.R. (2023). RNA Therapeutics. RNA.

[174] Zhang L., More K.R., Ojha A., Jackson C.B., Quinlan B.D., Li H., He W., Farzan M., Pardi N., Choe H. (2023). Effect of MRNA-LNP Components of Two Globally-Marketed COVID-19 Vaccines on Efficacy and Stability. Npj Vaccines.

[175] Titball R.W., Bernstein D.I., Fanget N.V.J., Hall R.A., Longet S., MacAry P.A., Rupp R.E., van Gils M., von Messling V., Walker D.H. (2024). Progress with COVID Vaccine Development and Implementation. Npj Vaccines.

[176] Witten J., Hu Y., Langer R., Anderson D.G. (2024). Recent Advances in Nanoparticulate RNA Delivery Systems. Proc. Natl. Acad. Sci. USA.

[177] Kim B., Hosn R.R., Remba T., Yun D., Li N., Abraham W., Melo M.B., Cortes M., Li B., Zhang Y. (2023). Optimization of Storage Conditions for Lipid Nanoparticle-Formulated Self-Replicating RNA Vaccines. J. Control Release.

[178] Meulewaeter S., Nuytten G., Cheng M.H.Y., De Smedt S.C., Cullis P.R., De Beer T., Lentacker I., Verbeke R. (2023). Continuous Freeze-Drying of Messenger RNA Lipid Nanoparticles Enables Storage at Higher Temperatures. J. Control Release.

[179] Ingle R.G., Fang W.-J. (2023). An Overview of the Stability and Delivery Challenges of Commercial Nucleic Acid Therapeutics. Pharmaceutics.

[180] Lamoot A., Lammens J., De Lombaerde E., Zhong Z., Gontsarik M., Chen Y., De Beer T.R.M., De Geest B.G. (2023). Successful Batch and Continuous Lyophilization of MRNA LNP Formulations Depend on Cryoprotectants and Ionizable Lipids. Biomater. Sci..

[181] Lin L., Su K., Cheng Q., Liu S. (2023). Targeting Materials and Strategies for RNA Delivery. Theranostics.

[182] Yan S., Na J., Liu X., Wu P. (2024). Different Targeting Ligands-Mediated Drug Delivery Systems for Tumor Therapy. Pharmaceutics.

[183] Kulkarni J.A., Witzigmann D., Chen S., Cullis P.R., van der Meel R. (2019). Lipid Nanoparticle Technology for Clinical Translation of SiRNA Therapeutics. Acc. Chem. Res..

[184] Pham T.T., Chen H., Nguyen P.H.D., Jayasinghe M.K., Le A.H., Le M.T. (2023). Endosomal Escape of Nucleic Acids from Extracellular Vesicles Mediates Functional Therapeutic Delivery. Pharmacol. Res..

[185] Narum S., Deal B., Ogasawara H., Mancuso J.N., Zhang J., Salaita K. (2024). An Endosomal Escape Trojan Horse Platform to Improve Cytosolic Delivery of Nucleic Acids. ACS Nano.

[186] Kim Y., Kim H., Kim E.H., Jang H., Jang Y., Chi S.-G., Yang Y., Kim S.H. (2022). The Potential of Cell-Penetrating Peptides for MRNA Delivery to Cancer Cells. Pharmaceutics.

[187] Sharma P., Hoorn D., Aitha A., Breier D., Peer D. (2024). The Immunostimulatory Nature of MRNA Lipid Nanoparticles. Adv. Drug Deliv. Rev..

[188] Elumalai K., Srinivasan S., Shanmugam A. (2024). Review of the Efficacy of Nanoparticle-Based Drug Delivery Systems for Cancer Treatment. Biomed. Technol..

[189] Aldosari B.N., Alfagih I.M., Almurshedi A.S. (2021). Lipid Nanoparticles as Delivery Systems for RNA-Based Vaccines. Pharmaceutics.

[190] Maruggi G., Zhang C., Li J., Ulmer J.B., Yu D. (2019). MRNA as a Transformative Technology for Vaccine Development to Control Infectious Diseases. Mol. Ther..

[191] Aldboush H.H.H., Ferdous M. (2023). Building Trust in Fintech: An Analysis of Ethical and Privacy Considerations in the Intersection of Big Data, AI, and Customer Trust. Int. J. Financ. Stud..

[192] Ouranidis A., Vavilis T., Mandala E., Davidopoulou C., Stamoula E., Markopoulou C.K., Karagianni A., Kachrimanis K. (2021). MRNA Therapeutic Modalities Design, Formulation and Manufacturing under Pharma 4.0 Principles. Biomedicines.

[193] Feng X., Su Z., Cheng Y., Ma G., Zhang S. (2023). Messenger RNA Chromatographic Purification: Advances and Challenges. J. Chromatogr. A.

[194] Kis Z. (2022). Stability Modelling of MRNA Vaccine Quality Based on Temperature Monitoring throughout the Distribution Chain. Pharmaceutics.

[195] Kumraj G., Pathak S., Shah S., Majumder P., Jain J., Bhati D., Hanif S., Mukherjee S., Ahmed S. (2022). Capacity Building for Vaccine Manufacturing Across Developing Countries: The Way Forward. Hum. Vaccin. Immunother..

[196] Mollocana-Lara E.C., Ni M., Agathos S.N., Gonzales-Zubiate F.A. (2021). The Infinite Possibilities of RNA Therapeutics. J. Ind. Microbiol. Biotechnol..

[197] Qu J., Nair A., Muir G.W., Loveday K.A., Yang Z., Nourafkan E., Welbourne E.N., Maamra M., Dickman M.J., Kis Z. (2024). Quality by Design for MRNA Platform Purification Based on Continuous Oligo-DT Chromatography. Mol. Ther.—Nucleic Acids.

[198] Whitley J., Zwolinski C., Denis C., Maughan M., Hayles L., Clarke D., Snare M., Liao H., Chiou S., Marmura T. (2022). Development of MRNA Manufacturing for Vaccines and Therapeutics: MRNA Platform Requirements and Development of a Scalable Production Process to Support Early Phase Clinical Trials. Transl. Res..

[199] Drago D., Foss-Campbell B., Wonnacott K., Barrett D., Ndu A. (2021). Global Regulatory Progress in Delivering on the Promise of Gene Therapies for Unmet Medical Needs. Mol. Ther. Methods Clin. Dev..

[200] Bak A., Friis K.P., Wu Y., Ho R.J.Y. (2019). Translating Cell and Gene Biopharmaceutical Products for Health and Market Impact. Product Scaling From Clinical to Marketplace: Lessons Learned and Future Outlook. J. Pharm. Sci..

[201] Rhym L.H., Anderson D.G. (2022). Nanoscale Delivery Platforms for RNA Therapeutics: Challenges and the Current State of the Art. Med.

[202] Padín-González E., Lancaster P., Bottini M., Gasco P., Tran L., Fadeel B., Wilkins T., Monopoli M.P. (2022). Understanding the Role and Impact of Poly (Ethylene Glycol) (PEG) on Nanoparticle Formulation: Implications for COVID-19 Vaccines. Front. Bioeng. Biotechnol..

[203] Kumari M., Acharya A., Krishnamurthy P.T. (2023). Antibody-Conjugated Nanoparticles for Target-Specific Drug Delivery of Chemotherapeutics. Beilstein J. Nanotechnol..

[204] Cox A., Lim S.A., Chung E.J. (2022). Strategies to Deliver RNA by Nanoparticles for Therapeutic Potential. Mol. Aspects Med..

[205] Lin Y., Chen X., Wang K., Liang L., Zhang H. (2024). An Overview of Nanoparticle-Based Delivery Platforms for MRNA Vaccines for Treating Cancer. Vaccines.

[206] Wong B., Birtch R., Rezaei R., Jamieson T., Crupi M.J.F., Diallo J.-S., Ilkow C.S. (2023). Optimal Delivery of RNA Interference by Viral Vectors for Cancer Therapy. Mol. Ther..

[207] Wang S., Liang B., Wang W., Li L., Feng N., Zhao Y., Wang T., Yan F., Yang S., Xia X. (2023). Viral Vectored Vaccines: Design, Development, Preventive and Therapeutic Applications in Human Diseases. Signal Transduct. Target. Ther..

[208] Bottens R.A., Yamada T. (2022). Cell-Penetrating Peptides (CPPs) as Therapeutic and Diagnostic Agents for Cancer. Cancers.

[209] Kravchenko S.V., Domnin P.A., Grishin S.Y., Zakhareva A.P., Zakharova A.A., Mustaeva L.G., Gorbunova E.Y., Kobyakova M.I., Surin A.K., Poshvina D.V. (2024). Optimizing Antimicrobial Peptide Design: Integration of Cell-Penetrating Peptides, Amyloidogenic Fragments, and Amino Acid Residue Modifications. Int. J. Mol. Sci..

[210] Dowaidar M. (2024). Uptake Pathways of Cell-Penetrating Peptides in the Context of Drug Delivery, Gene Therapy, and Vaccine Development. Cell. Signal..

[211] Nance K.D., Meier J.L. (2021). Modifications in an Emergency: The Role of N1-Methylpseudouridine in COVID-19 Vaccines. ACS Cent. Sci..

[212] Chernikov I.V., Ponomareva U.A., Chernolovskaya E.L. (2023). Structural Modifications of SiRNA Improve Its Performance In Vivo. Int. J. Mol. Sci..

[213] Parveen S., Gupta P., Kumar S., Banerjee M. (2023). Lipid Polymer Hybrid Nanoparticles as Potent Vehicles for Drug Delivery in Cancer Therapeutics. Med. Drug Discov..

[214] Niculescu A.-G., Bîrcă A.C., Grumezescu A.M. (2021). New Applications of Lipid and Polymer-Based Nanoparticles for Nucleic Acids Delivery. Pharmaceutics.

[215] Wei P.-S., Thota N., John G., Chang E., Lee S., Wang Y., Ma Z., Tsai Y.-H., Mei K.-C. (2024). Enhancing RNA-Lipid Nanoparticle Delivery: Organ- and Cell-Specificity and Barcoding Strategies. J. Control Release.

[216] AboulFotouh K., Southard B., Dao H.M., Xu H., Moon C., Williams III R.O., Cui Z. (2024). Effect of Lipid Composition on RNA-Lipid Nanoparticle Properties and Their Sensitivity to Thin-Film Freezing and Drying. Int. J. Pharm..

[217] Cheng X., Xie Q., Sun Y. (2023). Advances in Nanomaterial-Based Targeted Drug Delivery Systems. Front. Bioeng. Biotechnol..

[218] Zhou H., Chen D.S., Hu C.J., Hong X., Shi J., Xiao Y. (2023). Stimuli-Responsive Nanotechnology for RNA Delivery. Adv. Sci..

[219] Webb C., Ip S., Bathula N.V., Popova P., Soriano S.K.V., Ly H.H., Eryilmaz B., Nguyen Huu V.A., Broadhead R., Rabel M. (2022). Current Status and Future Perspectives on MRNA Drug Manufacturing. Mol. Pharm..

[220] Cheng F., Wang Y., Bai Y., Liang Z., Mao Q., Liu D., Wu X., Xu M. (2023). Research Advances on the Stability of MRNA Vaccines. Viruses.

[221] Liu A., Wang X. (2022). The Pivotal Role of Chemical Modifications in MRNA Therapeutics. Front. Cell Dev. Biol..

[222] Han G., Noh D., Lee H., Lee S., Kim S., Yoon H.Y., Lee S.H. (2023). Advances in MRNA Therapeutics for Cancer Immunotherapy: From Modification to Delivery. Adv. Drug Deliv. Rev..

[223] Liu X., Zhang Y., Zhou S., Dain L., Mei L., Zhu G. (2022). Circular RNA: An Emerging Frontier in RNA Therapeutic Targets, RNA Therapeutics, and MRNA Vaccines. J. Control Release.

[224] Papukashvili D., Rcheulishvili N., Liu C., Ji Y., He Y., Wang P.G. (2022). Self-Amplifying RNA Approach for Protein Replacement Therapy. Int. J. Mol. Sci..

[225] Zhou W., Jiang L., Liao S., Wu F., Yang G., Hou L., Liu L., Pan X., Jia W., Zhang Y. (2023). Vaccines’ New Era-RNA Vaccine. Viruses.

[226] Taibi T., Cheon S., Perna F., Vu L.P. (2024). MRNA-Based Therapeutic Strategies for Cancer Treatment. Mol. Ther..

[227] Du Y., Liu Y., Hu J., Peng X., Liu Z. (2023). CRISPR/Cas9 Systems: Delivery Technologies and Biomedical Applications. Asian J. Pharm. Sci..

[228] Richardson C., Kelsh R.N., Richardson R.J. (2023). New Advances in CRISPR/Cas-Mediated Precise Gene-Editing Techniques. Dis. Model. Mech..

[229] Lin M.J., Svensson-Arvelund J., Lubitz G.S., Marabelle A., Melero I., Brown B.D., Brody J.D. (2022). Cancer Vaccines: The next Immunotherapy Frontier. Nat. Cancer.

[230] Singh S., Khasbage S., Kaur R.J., Sidhu J.K., Bhandari B. (2022). Chimeric Antigen Receptor T Cell. Indian J. Pharmacol..

[231] Pozzi D., Caracciolo G. (2023). Looking Back, Moving Forward: Lipid Nanoparticles as a Promising Frontier in Gene Delivery. ACS Pharmacol. Transl. Sci..

[232] Gajbhiye K.R., Salve R., Narwade M., Sheikh A., Kesharwani P., Gajbhiye V. (2023). Lipid Polymer Hybrid Nanoparticles: A Custom-Tailored next-Generation Approach for Cancer Therapeutics. Mol. Cancer.

[233] Peng X., Fang J., Lou C., Yang L., Shan S., Wang Z., Chen Y., Li H., Li X. (2024). Engineered Nanoparticles for Precise Targeted Drug Delivery and Enhanced Therapeutic Efficacy in Cancer Immunotherapy. Acta Pharm. Sin. B.

[234] Han X., Mitchell M.J., Nie G. (2020). Nanomaterials for Therapeutic RNA Delivery. Matter.

[235] Nash J.A., Manning M.D., Gulyuk A.V., Kuznetsov A.E., Yingling Y.G. (2022). Gold Nanoparticle Design for RNA Compaction. Biointerphases.

[236] Lv T., Meng Y., Liu Y., Han Y., Xin H., Peng X., Huang J. (2023). RNA Nanotechnology: A New Chapter in Targeted Therapy. Colloids Surfaces B Biointerfaces.

[237] Lima E.S., dos Santos D., Souza A.L., Macedo M.E., Bandeira M.E., Junior S.S.S., Fiuza B.S.D., Rocha V.P.C., dos Santos Fonseca L.M., Nunes D.D.G. (2023). RNA Combined with Nanoformulation to Advance Therapeutic Technologies. Pharmaceuticals.

[238] Naimi N., Seyedmirzaei H., Hassannejad Z., Soltani Khaboushan A. (2024). Advanced Nanoparticle Strategies for Optimizing RNA Therapeutic Delivery in Neurodegenerative Disorders. Biomed. Pharmacother..

[239] Halloy F., Biscans A., Bujold K.E., Debacker A., Hill A.C., Lacroix A., Luige O., Strömberg R., Sundstrom L., Vogel J. (2022). Innovative Developments and Emerging Technologies in RNA Therapeutics. RNA Biol..

[240] Thi H.V., Hoang T.-N., Le N.Q.K., Chu D.-T. (2024). Application of Data Science and Bioinformatics in RNA Therapeutics. Prog. Mol. Biol. Transl. Sci..

[241] Banwait J.K., Bastola D.R. (2015). Contribution of Bioinformatics Prediction in MicroRNA-Based Cancer Therapeutics. Adv. Drug Deliv. Rev..

[242] Alowais S.A., Alghamdi S.S., Alsuhebany N., Alqahtani T., Alshaya A.I., Almohareb S.N., Aldairem A., Alrashed M., Bin Saleh K., Badreldin H.A. (2023). Revolutionizing Healthcare: The Role of Artificial Intelligence in Clinical Practice. BMC Med. Educ..

[243] Hwang H., Jeon H., Yeo N., Baek D. (2024). Big Data and Deep Learning for RNA Biology. Exp. Mol. Med..

[244] Chatterjee S., Bhattacharya M., Lee S.-S., Chakraborty C. (2023). An Insight of Different Classes of RNA-Based Therapeutic, Nanodelivery and Clinical Status: Current Landscape. Curr. Res. Biotechnol..

